# Is the impact of childhood influenza vaccination less than expected: a transmission modelling study

**DOI:** 10.1186/s12879-017-2344-6

**Published:** 2017-04-11

**Authors:** Felix Weidemann, Cornelius Remschmidt, Silke Buda, Udo Buchholz, Bernhard Ultsch, Ole Wichmann

**Affiliations:** 1 Immunization Unit, Robert Koch-Institute, Seestr. 10, 13359 Berlin, Germany; 2 Respiratory Disease Unit, Robert Koch-Institute, Seestr. 10, 13359 Berlin, Germany

**Keywords:** Influenza, childhood vaccination, transmission model, NNV, Bayesian inference

## Abstract

**Background:**

To reduce the burden of severe influenza, most industrialized countries target specific risk-groups with influenza vaccines, e.g. the elderly or individuals with comorbidities. Since children are the main spreaders, some countries have recently implemented childhood vaccination programs to reduce overall virus transmission and thereby influenza disease in the whole population. The introduction of childhood vaccination programs was often supported by modelling studies that predicted substantial incidence reductions. We developed a mathematical transmission model to examine the potential impact of childhood influenza vaccination in Germany, while also challenging established modelling assumptions.

**Methods:**

We developed an age-stratified SEIR-type transmission model to reproduce the epidemic influenza seasons between 2003/04 and 2013/14. The model was built upon German population counts, contact patterns, and vaccination history and was fitted to seasonal data on influenza-attributable medically attended acute respiratory infections (I-MAARI) and strain distribution using Bayesian methods. As novelties we (i) implemented a stratified model structure enabling seasonal variability and (ii) deviated from the commonly assumed mass-action-principle by employing a phenomenological transmission rate.

**Results:**

According to the model, by vaccinating primarily the elderly over ten seasons 4 million (95% prediction interval: 3.84 – 4.19) I-MAARI were prevented which corresponds to an 8.6% (8.3% – 8.9%) reduction compared to a no-vaccination scenario and a number-needed-to-vaccinate (NNV) to prevent one I-MAARI of 37.1 (35.5 – 38.7). Additional vaccination of 2-10 year-old children at 40% coverage would have led to an overall I-MAARI reduction of 17.8% (17.1 – 18.7%) mostly due to indirect effects with a NNV of 20.7 (19.6 – 21.6). When employing the traditional mass-action-principle, the model predicted a more than 3-fold higher I-MAARI reduction (55.6%) due to childhood vaccination.

**Conclusion:**

In Germany, the introduction of routine childhood influenza vaccination could considerably reduce I-MAARI among all age-groups and improve the NNV. However, the predicted impact is much lower compared to previous studies, which is primarily caused by our phenomenological approach to modelling influenza virus transmission.

**Electronic supplementary material:**

The online version of this article (doi:10.1186/s12879-017-2344-6) contains supplementary material, which is available to authorized users.

## Background

Around the globe, annual influenza epidemics result in considerable morbidity and mortality in all parts of the population. Children have the highest attack rate [1, 2], but the elderly and people with comorbidities are at particular risk for severe influenza disease and influenza-associated complications [3]. Therefore, Germany and many other industrialized countries recommend annual vaccination of these at-risk groups focusing on the direct benefits of the vaccine [4].

However, since the effectiveness of influenza vaccines is limited in these at-risk groups [5, 6] and because children are considered the primary spreaders of influenza, childhood vaccination programs might reduce influenza disease burden not only in the vaccinated but also in the non-vaccinated population through indirect effects [7]. In the UK, for example, routine vaccination of all children aged two to 17 years is recommended in addition to the vaccination of risk-groups. In the US, annual vaccination is recommended for all people above 6 months of age [8]. According to impact results, the UK childhood vaccination program caused considerable indirect effects also among adults [9]. Field studies from the USA suggested that indirect effects are very pronounced in closed communities [10]. However, the indirect impact of school vaccination programs on US county level turned out to be low [11, 12].

The availability on new live attenuated influenza vaccines (LAIV) also supported the introduction of childhood vaccination, as LAIV promised higher vaccine efficacy (VE) among children and easier administration compared to inactivated vaccines [13]. Recent results though question the beneficial effectiveness of LAIV [14], which led to withdrawal of LAIV recommendation in the US [15].

To predict the potential impact of including a new vaccine or implementing a new strategy in the national immunization program, many national decision makers utilize mathematical transmission modelling prior to introduction. For influenza, every transmission model published as of today predicted considerable population-wide case reductions due to childhood immunization programs, which contrasts the heterogeneous impact of vaccination observed in the field [16–19].

To explore explanations for the partial discrepancy between modelled and observed childhood vaccination impact, we propose a new modelling approach of stratification by season and subtype to handle variability of observed influenza epidemics. Moreover, we challenge established assumptions regarding the modelling of contact pattern and force of infection. Using Bayesian methods, our employed transmission model is calibrated through estimated numbers of medically-attended influenza cases in Germany and virological data on the yearly distribution of influenza subtypes [20].

The objective of our work was to develop a transmission model that is capable of reproducing the influenza epidemics in Germany observed over the past 10 years. Using the model we estimated the number of medically-attended influenza cases that could have been prevented by a childhood vaccination strategy in Germany, which provides necessary evidence to support immunization decision making.

## Methods

We developed an age-stratified SEIR-type transmission model to reproduce the epidemic influenza seasons between 2003/04 and 2013/14 excluding the pandemic season 2009/10. The model was based on German population counts and vaccination history and fitted to seasonal data on influenza excess consultations and strain distribution as described below. Here, we give an overview on the most relevant model aspects whereas full details are provided in the supplemental material (see Additional file 1).

### Epidemiological data

#### Influenza surveillance and virological data

The German working group on influenza (Arbeitsgemeinschaft Influenza, AGI) operates a syndromic surveillance system using a GP sentinel network [20]. On average 600 participating practitioners provide weekly reports on the frequency of medically-attended acute respiratory infections (MAARI) for five age groups: 0-4, 5-14, 15-34, 35-59, and 60+ years of age.

The season and age-group specific number of influenza-attributable MAARI (I-MAARI) are estimated through a time series approach based on the reported MAARI incidence [20].

The National Influenza Reference Center provided data of the virological surveillance of the AGI including seasonal distribution over the influenza types A(H1N1), A(H3N2) and B. Here, A(H1N1) refers either to the pandemic variant A(H1N1)pdm09 or to the pre-pandemic variant A(H1N1)prepan, depending on season. Since the season 2008/09 B-positive samples are further divided into B-Yamagata and B-Victoria yielding overall four different subtypes. Although inaccurate, from here on we use *subtype* to refer to the two A subtypes and the two B-lineages. For seasons prior to 2008/09 with missing lineage information we identified the circulating B-lineages from the annual AGI reports [21]. For the epidemic season 2005/06 that exhibited considerable co-circulation with 90% B-Victoria and 10% B-Yamagata, we randomly assigned each B-positive sample to one of the two lineages according to the B-lineage distribution given in the AGI report [22].

#### Vaccination data

In Germany during the modelled time horizon, influenza vaccination was primarily recommended for at-risk groups, i.e. people aged 60+ years and individuals with comorbidities [23].

To reproduce the past vaccination history, we obtained vaccine coverage rates for each of the included seasons from health insurance claims data as described by Rieck et al. [24]. The seasonal coverage was highest among elderly over 60 years with on average 44.2 % between 2003/2004 and 2013/2014. The mean coverage among children (0-19 years) and adults under 60 years (20-59 years) was 5.4% and 9.7%, respectively.

For the majority of modelled seasons only trivalent inactivated vaccines (TIV) from various manufacturers were administered in Germany, although in recent years also quadrivalent vaccines (QIV) and for children LAIV became available. Since market share of QIV and LAIV was small in Germany (data obtained from Insight Health GmbH & Co. KG) and information on the type/brand of administered influenza vaccine is not included in the insurance claims data, we assumed one generic trivalent vaccine to be applied in the model.

Because influenza viruses change dynamically over time (antigenic drift) and the vaccine is reformulated every season according to recommendations from the World Health Organization (WHO), the vaccine effectiveness (VE) may differ from season to season, by subtype, and by age. Thus, the VE in our model also distinguished between subtypes, seasons, and three age groups: 0-14, 15-59, and 60+ years. For VE estimates of past seasons we relied on data from the European I-MOVE network (Influenza - Monitoring Vaccine Effectiveness), Cochrane reviews, and AGI reports [25–32]. For each season since 2008/2009, we specified the model VE based on I-MOVE that assessed country-, subtype-, and age-specific VE estimates [27–32]. We prioritized I-MOVE estimates that were adjusted for study site, chronic condition, and other influential factors. Where I-MOVE results were not available, we used estimates from Cochrane reviews that measured VE in both adults and children for well-matched and poorly-matched seasons [25, 26]. Information on seasonal matching was taken from the annual AGI reports.

Although TIV contains only one B-lineage component, some clinical studies detected a cross-protection for the B-lineage that was not included in the vaccine, which was measured at 60% compared to the VE against the included B-lineage [33, 34]. Thus, for the B-lineage not included in TIV according to WHO recommendation we assumed this 60% cross-protection as already done in previous models [35]. Combining the observed seasonal B-lineage mix with the overall B-lineage VE estimates then yields VE estimates specific to each B-lineage.

### Transmission model

#### Model structure

The season and subtype specific transmission dynamics are captured through an age-structured SEIR model introduced by Vynnycky et al. and revisited elsewhere [16–19, 35–37]. The model divides each age-group into susceptibles (*S*), latently infected but not yet infectious individuals (*E*), infectious (*I*), and recovered individuals (*R*). These are further split into vaccinated and unvaccinated individuals which yields in total eight age-stratified model compartments.

During an epidemic season and for each subtype, susceptible people might acquire infection (*S → E*), become infectious (*E → I*), and upon recovery (*I → R*) become immune for the remainder of the season. These dynamics are formulated as a system of ordinary differential equations.

In each season only a fraction of the population starts as susceptible since initial immunity could be inherited from prior infections or established through vaccination. Here, we assume an all-or-nothing vaccination effect [38]. Waning of vaccine protection during the season is not taken into account, but the protection is assumed to vanish completely at the end of each season.

We pursue a stratified modelling approach such that each subtype and season is embedded within a global parametric framework [39]. Hence, although the transmission dynamics of each subtype within each season are modelled separately, these single models share not only the same structure but also certain parameter values that are likely unaffected by season and subtype, e.g. contact patterns and consultation rates. Conversely, those transmission aspects that are presumably specific to either season or subtype, and thus are causing the observed seasonal variability, are allowed to differ accordingly. See Table 1 for a list of all model parameters and their corresponding stratification.

**Table 1:** Model parameters to be estimated from epidemiological data, their prior ranges and posterior estimates

Parameter	Interpretation	Stratification	Prior domain	Posterior estimate (95% CrI^a^)	Source
*γ*	Recovery rate (inverse infectious duration)	None	1/*γ* ∈ [1/7; 2.5/7]	2.85 [2.81; 2.91]	[19, 49]
*R*_*e*_	Baseline transmission rate	none	[0; 1]	0.13 [0.10; 0.15]	Assumption
*λ*_*o*_	External force of infection	None	[0 ; ∞ )	1.49 x 10^-8^ [0.96 x 10^-8^; 2.15 x 10^-8^]	Assumption
*ρ*	Spatial clustering parameter	None	[0; 1]	0.76 [0.75; 0.78]	[40]
*m*	Contact matrix mixing parameter	None	[1 ; ∞ )	1.04 [1.01; 1.09]	[48]
*δ*	Amplitude of transmission rate	None	[0 ; ∞ )	2.38 [2.23; 2.53]	Assumption
${p}_a^{(c)}$	Age specific medical consultation probability	None	${p}_{<5}^{(c)}\in \left[0.28;0.46\right]$ ${p}_{\ge 5}^{(c)}\in \left[0.19;0.38\right]$	0.453 [0.446; 0.457] 0.373 [0.365; 0.377]	[55]
*t*_*s*_	Seasonal shift in peak transmission	By season	[−0.125; 0.125]	See Additional file 1	Assumption
*t*_*z*_	Subtype-specific shift in peak transmission	By subtype	[−0.5; 0.5]	See Additional file 1	Assumption
*σ*_*a*_	Age specific susceptibility	By subtype	[0; 1]	See Fig. 2	[52]
*φ*	Season specific susceptible fraction	By season and subtype	[0; 1]	See Fig. 2	[52]

^a^ credibility interval

#### Susceptible Population

The susceptible fraction of the model population may differ with respect to each subtype and season, since varying susceptibility is assumed to be the primary cause for variability within seasonal influenza epidemics. Within our model, the susceptible fraction for each subtype-season is controlled by two aspects: (i) an age-specific susceptibility *σ*_*a*_ accounting for increased subtype-specific immunity within the two age-groups 15-59 and 60+ years, and (ii) a season-specific factor *φ* that additionally shrinks the subtype-specific susceptible population fraction due to immunity gained within recently past seasons.

#### Disease transmission

For each age-group *i* the force of infection *λ*_*i*_ results from the age dependent contact behavior, the prevalence of infection, its transmissibility, and a constant risk *λ*_*o*_ to import influenza infection from outside of the German population.

1 $$ {\lambda}_i\left( t, I(t)\right)={R}_e\mathit{\exp}\left\{\delta\ \mathit{\sin}\left(2\pi \left(\frac{t}{52}-{t}_z+{t}_s\right)\right)\right\}\ \sum_{j=1}^{n_a}{\beta}_{i, j}^{(eff)}{\left(\frac{I_j(t)}{N_j(t)}\right)}^{\rho}+{\lambda}_o $$

In contrast to implementing the mass-action-principle that assumes spatial homogeneity and is commonly used for pathogen transmission modelling, we apply a phenomenological transmission rate that enables a dampened growth of the force of infection for an increasing prevalence subject to the power parameter ρ ≤ 1 [40, 41]. This transmission rate is motivated by effects which lead to a declining effective reproduction rate, such as reactive behavior changes during an epidemic or a potential spatial clustering of the infection resulting from the non-homogenous spread within a population [42]. In our model, the infectious pressure *λ*_*i*_ applicable to susceptibles in age group *i* therefore increases concavely, i.e. at a decreasing marginal rate, with the infections prevalence in each age group *j* (given by the number of infectious people *I*_*j*_(*t*) divided by the population size *N*(*t*)_*j*_, *j* = 1 ,  …  , *n*_*a*_). This concaveness is more pronounced for small values of ρ [43].

The contact rates ${\beta}_{i, j}^{(eff)}$ denote the average number of effective contacts from individuals in age- group *i* with individuals in age group *j* that are – according to the social contact hypothesis – proportional to the number of social contacts between these age group [44]. We estimated social contact frequencies based on the German part of the POLYMOD survey using a spline regression approach [44, 45]. We restricted the data to contacts of physical nature or of at least 15 minutes duration as these were found to be a good proxy for contacts likely leading to transmission of seasonal influenza [36, 46].

However, the POLYMOD study surveyed the behavior of primarily healthy people. A contact survey conducted throughout the pandemic season in 2009/2010 measured the social activity of symptomatically ill people and once again after their recovery [47]. Based on this data it was shown that ill people have a higher number of household contacts whereas school and work contacts are less frequent [48]. We utilized these results to estimate a contact matrix ***β*** ^(*sick*)^ subject to symptomatic illness. The effective contact matrix was then modelled to be a weighted combination of the two contact matrices for healthy and sick people.

2 $$ {\boldsymbol{\beta}}^{(eff)}={\boldsymbol{\beta}}^{(healthy)}+ m\times {\boldsymbol{\beta}}^{(sick)} $$

The weighting parameter *m* > 1 secures that the contact matrix subject to ill people has a bigger impact due to the high probability for developing symptoms, but also because symptomatic people have a higher infectiousness [48].

The parameter *R*_*e*_ controls the transmissibility of the infection whereas *z*(*t*) reflects the intra-seasonal variation of the transmissibility as one oscillation over the season.

3 $$ z(t)=\mathit{\exp}\left\{\delta\ \mathit{\sin}\left(2\pi \left(\frac{t}{52}-{t}_z+{t}_s\right)\right)\right\} $$

The parameter *t*_*z*_ and *t*_*s*_ control the timely shift of peak transmission with respect to subtype and season, respectively, and thus may vary accordingly. The magnitude of the seasonal oscillation is controlled by *δ*.

An overview on each parameters interpretation and potential stratification by season or subtype is given in Table 1.

### Parameter estimation

Parameters that are well supported through data such as demographics and vaccination history are kept fixed within the model. Model parameters based on weak evidence were estimated within a Bayesian framework using the available disease burden data (see Table 1).

Prior distributions and plausible ranges for all estimated parameters were defined based on literature if available. Additionally, the likelihood function specified below measures the plausibility of the two data sets, I-MAARI data and virological data, subject to the model.

The number of I-MAARI ${D}_{t, a}^{(s)}$ per week *t* and age-group *a* determines for each season *s* the magnitude of the influenza wave as predicted by the model that is the aggregation of the four subtype specific waves. Thus, let ${X}_{t, a}^{\left( z, s\right)}\left(\boldsymbol{\theta} \right)$ denote the model predicted number of influenza cases due to subtype *z* and subject to the parameter vector ***θ***. The predicted number of I-MAARI is then given by

4 $$ {Y}_{t, a}^{(s)}\left(\boldsymbol{\theta} \right)=0.67\times {p}_a^{(c)}\left({X}_{t, a}^{\left( AH1 N1, s\right)}\left(\boldsymbol{\theta} \right)+{X}_{t, a}^{\left( AH3 N2, s\right)}\left(\boldsymbol{\theta} \right)+{X}_{t, a}^{\left( B- Yam, s\right)}\left(\boldsymbol{\theta} \right)+{X}_{t, a}^{\left( B- Vic, s\right)}\left(\boldsymbol{\theta} \right)\right) $$

Where 0.67 gives the probability for developing symptoms [49] and ${p}_a^{(c)}$ refers to the age specific probability for seeking medical treatment, i.e. the consultation rate due to influenza. The probability for developing symptoms was chosen to be constant in order to secure identifiability of the model. The number of I-MAARI estimated by AGI is then assumed to be negative-binomially distributed with expectation ${Y}_{t, a}^{(s)}\left(\boldsymbol{\theta} \right)$ and dispersion *d*_*t*_, i.e.

5 $$ L\left(\boldsymbol{D}|\boldsymbol{\theta} \right)=\prod_{t\in T\ }\prod_{s\in S\ }\prod_{a\in A\ } NegBin\left({D}_{t, a}^{(s)}|{Y}_{t, a}^{(s)}\left(\boldsymbol{\theta} \right),{d}_t\right) $$

The subtype distribution is governed through the virological data containing the weekly number of positive tests ${P}_{t, a}^{\left( z, s\right)}$ for each subtype *z*. We constructed the likelihood for observing the subtype distribution ${\boldsymbol{P}}_{t, a}^{(s)}=\left({P}_{t, a}^{\left( AH1 N1, s\right)},{P}_{t, a}^{\left( AH3 N2, s\right)},{P}_{t, a}^{\left( B- Yam,\kern0.5em  s\right)},{P}_{t, a}^{\left( B- Vic, s\right)}\right)$ by assuming that each influenza case has the same probability of leading to a positive virological test and identification that leads to a Dirichlet-multinomial likelihood.

6 $$ L\left(\boldsymbol{P}|\boldsymbol{\theta} \right)=\prod_{t\in T\ }\prod_{s\in S\ }\prod_{a\in A\ } DirichMult\left({\boldsymbol{P}}_{t, a}^{(s)}|\left({X}_{t, a}^{\left( AH1 N1, s\right)},{X}_{t, a}^{\left( AH3 N2, s\right)},{X}_{t, a}^{\left( B- Yam, s\right)},{X}_{t, a}^{\left( B- Vic, s\right)}\right)\right) $$

The posterior distribution is then derived as the product of the prior and the two likelihood functions *L*(***D***| ***θ***) and *L*(***P***| ***θ***) assuming conditional independence. A sample from the posterior distribution is obtained by applying adaptive Markov chain Monte Carlo sampling as done previously [16, 50].

### Investigated model scenarios

We developed alternative model scenarios to investigate the sensitivity of the respective results caused by different assumptions. Alternative assumptions included assuming no B-lineage cross-protection (S1), switching from phenomenological transmission to mass-action-transmission (S2), applying the contact matrix from only healthy individuals (S3), and disabling any indirect effect (S4). The fit of each model was measured through its respective marginal likelihood of the data [51].

### Vaccination impact analysis

To assess the impact of the past vaccination program (historic vaccination) on the number of prevented I-MAARI we simulated a scenario without vaccination. The potential impact of routine childhood vaccination was estimated by simulating a scenario with increased vaccination coverage of 40% among all children from 2 to 10 years additional to the historic vaccination. Furthermore, we varied the target group to include children up to 17 years or only up to 6 years and also examined alternative childhood coverage rates of 20% or 60%. The potential effect of QIV was examined by assuming 100% B-lineage cross protection. Although the beneficial effect of LAIV is uncertain, in order to investigate the potential impact of an overall more effective influenza vaccine we assumed LAIV having a 50% higher VE among children age 2 to 6 years compared to TIV. We also investigated a scenario of increased coverage among elderly over 60 years. The impact of each scenario was measured as the I-MAARI reduction in comparison to the scenario without vaccination.

## Results

### Model fitting

**Fig. 1:**
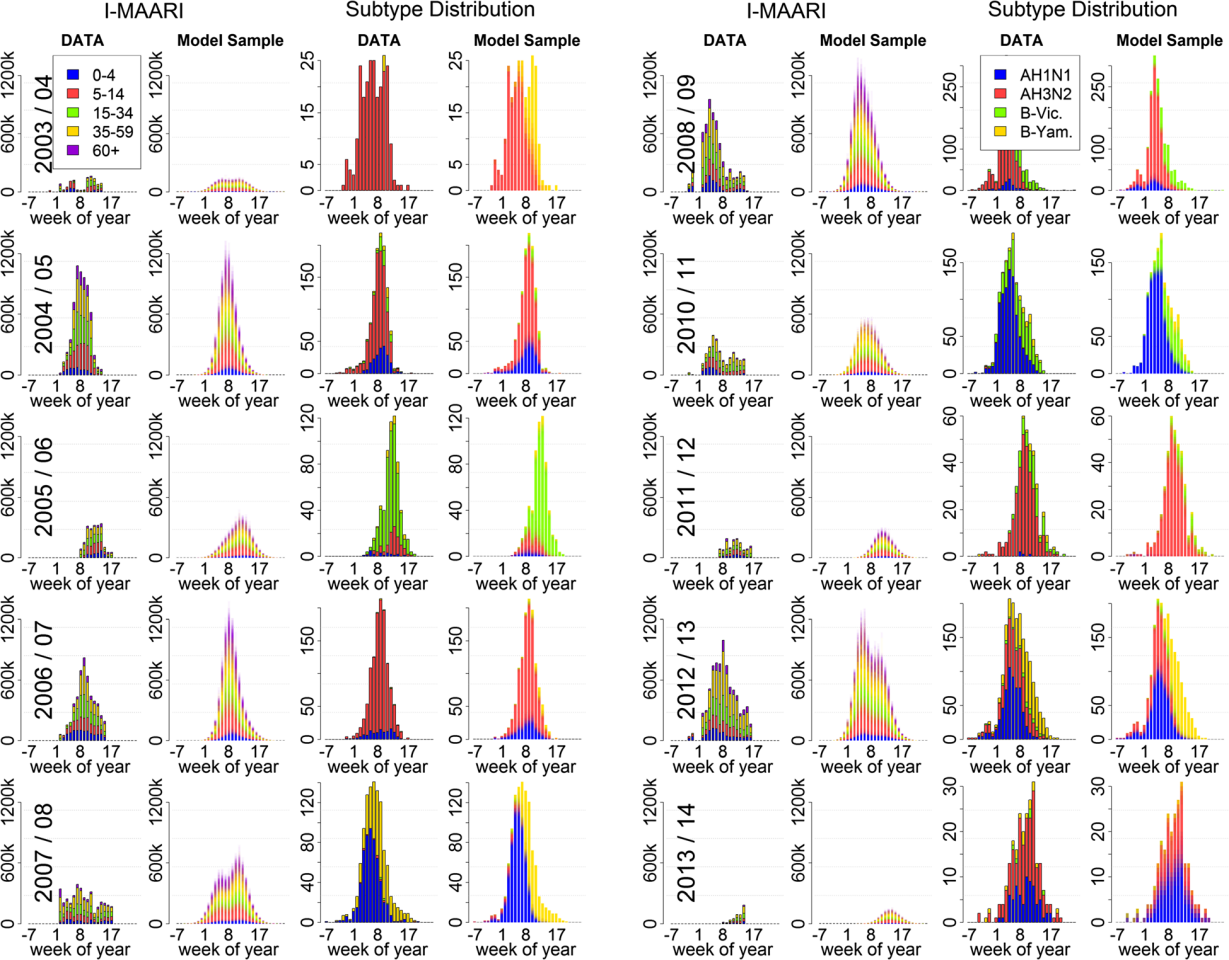
Seasonal influenza data and output from the fitted transmission model. Influenza epidemics according to data and fitted model for the seasons 2003/04 till 2013/14 excluding the pandemic season 2009/10. The first and second column show the I-MAARI estimated by AGI and NIC virological data, respectively. The third and fourth column provide corresponding model-predicted consultation numbers and subtype distribution based on overlaid output subject to 1000 parameter vectors drawn from the posterior distribution

The ten reproduced influenza waves are shown in Fig. 1. The I-MAARI curves including their magnitude, peak time, and age distribution are reflected by the model-predicted waves. The distribution and timely occurrence of the single subtypes are well reproduced, with the only exception being the 2003/04 season for which the model predicts the circulation of also the B-Yamagata lineage. Corresponding parameter estimates are displayed in Table 1.

**Fig. 2:**
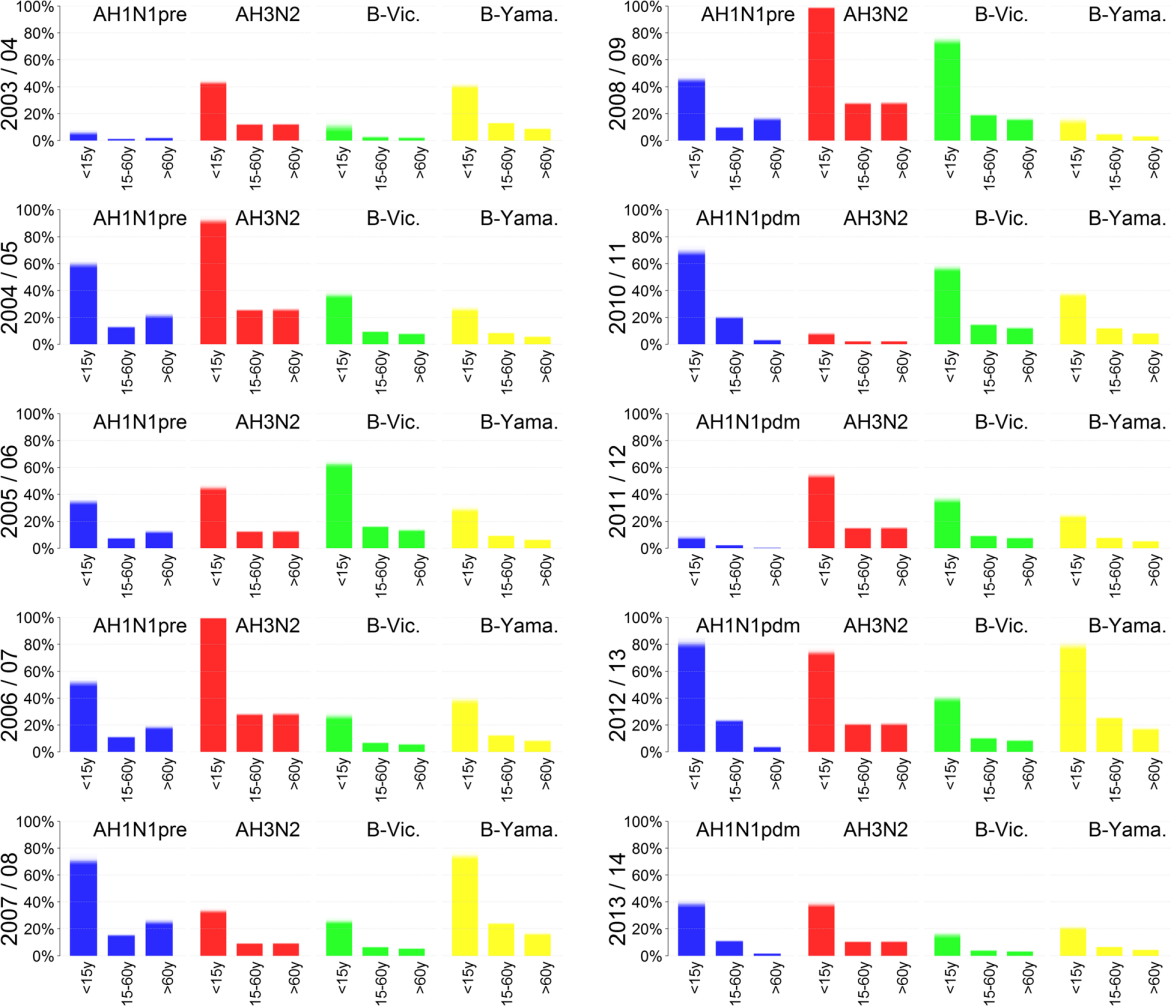
Susceptible population fractions. Posterior estimates for the seasonal subtype specifc susceptibility profiles determined through the parameters *σ*_*a*_ and *φ*. The estimates are displayed as the overlaid fractions according to 1000 draws from the posterior

The estimated susceptible population fractions given in Fig. 2 vary considerably by season and subtype. For all subtypes the susceptible fraction among children was estimated to be much larger compared to older age groups. Moreover, the overall susceptible population fraction with respect to one single subtype, especially AH1N1, can vary considerably for subsequent seasons. An overall smaller population fraction was susceptible to the B-lineages as compared to influenza A. Comparing prepandemic and pandemic A(H1N1), a smaller fraction of people over 60 years was susceptible to the pandemic strain A(H1N1)pdm09, whereas in the age group 15 to 60 years a larger fraction is susceptible to A(H1N1)pdm09.

### Childhood vaccination impact

**Fig. 3:**
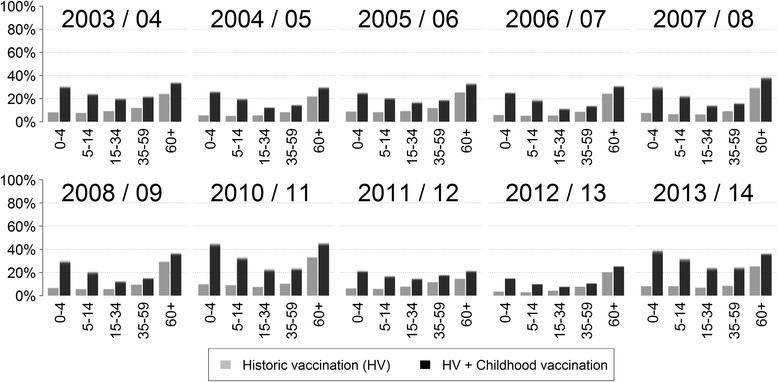
Relative decrease of I-Maari by season. Predicted relative I-MAARI reduction for each age group and season due to (i) the historic vaccination program and (ii) an alternative vaccination scenario additionally targeting children aged 2 to 10 years at 40% vaccination (based on 1000 draws from the posterior). The reduction is measured against a hypothetical scenario without influenza vaccination, respectively

Across the ten modelled seasons the historic vaccination program targeting only at-risk groups has prevented 8.6% of all I-MAARI that would have occurred in a scenario without vaccination as predicted by the model (Table 2). An additional vaccination uptake to 40% among 2 to 10 year old children could have reduced the influenza disease burden by overall 17.8% compared to a scenario without vaccination, which corresponds to an average 828,000 prevented I-MAARI annually. Within the childhood vaccination scenario, the relative reduction per season ranged from 10.6% to 27.2% (Fig. 3). It was most pronounced among elderly with a mean I-MAARI reduction of 31.0%, followed by children with 25.1% and 18.5% prevented I-MAARI in the age groups <5 years and 5-14 years, respectively. I-MAARI incidence among adults was predicted to be reduced by 12.1% and 14.5% for the age groups 15 to 34 years and 35 to 60 years, respectively. Extending the hypothetical childhood vaccination to children aged 2 to 17 years increased the overall I-MAARI reduction to 22.9% whereas restricting childhood vaccination to children aged 2 to 6 years yields a reduction of 14.3% compared to a no vaccination scenario (Table 2). Administration of live attenuated vaccines for children or utilization of quadrivalent vaccines within the base childhood vaccination scenario (2-10 years, 40% coverage) would have led to an additional reduction of 3.8% or 2.3%, respectively. Increasing the coverage among elderly over 60 years to 100% would reduce I-MAARI by 12.1% compared to no vaccination.

**Table 2:** Predicted impact of different vaccination scenarios assuming different target groups and coverage rates

Vaccination scenario	Relative reduction^a^ of I-MAARI (with 95%-PI^b^)			Absolute reduction^a^ of I-MAARI in million (with 95%-PI^b^)			NNV^c^ to prevent one I-MAARI		
Historic vaccination scenario	8.6% (8.3% – 8.9%)			-4.00 (-3.84 – -4.19)			37.1 (35.5 – 38.7)		
	Vaccine coverage for childhood vaccination								
	20%	40%	60%	20%	40%	60%	20%	40%	60%
Historic vaccination rates and 2 – 6 year old children	11.0% (10.6% – 11.5%)	14.3% (13.6% – 14.9%)	17.4% (16.6% – 18.2%)	-5.12 (-4.89 – -5.36)	-6.62 (-6.32 – -6.97)	-8.07 (-7.67 – -8.46)	30.0 (28.7 – 31.4)	24.3 (23.1 – 25.5)	20.8 (19.9 – 21.9)
Historic vaccination rates and 2 – 10 year old children	12.5% (12.0% – 13.2%)	17.8% (17.1% – 18.7%)	23.0% (21.9% – 24.1)	-5.82 (-5.52 – -6.11)	-8.28 (-7.91 – -8.75)	-10.67 (-10.17 – -11.29)	27.2 (25.8 – 28.6)	20.7 (19.6 – 21.6)	17.3 (16.3 – 18.1)
Historic vaccination rates and 2 – 17 year old children	14.7% (14.0% – 15.4%)	22.9% (21.8% – 24.1%)	30.7% (29.3% – 32.0%)	-6.80 (-6.50 – -7.13)	-10.59 (-10.08 – -11.16)	-14.23 (-13.54 – -15.00)	24.5 (23.3 – 25.6)	18.1 (17.1 – 19.0)	15.2 (14.4 – 15.9)
Historic vaccination and 2 – 10 year old children (40% coverage) using quadrivalent vaccines.	19.4% (18.6% – 20.4%)			-9.00 (-8.56 – -9.50)			19.0 (18.0 – 20.0)		
Historic vaccination and 2 – 10 year old children (40% coverage) using LAIV^d^.	20.9% (19.9% – 21.9%)			-9.70 (-9.23 – -10.15)			17.7 (16.8 – 18.6)		
Historic vaccination with complete coverage (100%) among elderly (≥ 60 years)	12.1% (11-6% – 12.4%)			-5.62 (-5.38 – -5.87)			47.6 (45.6 – 49.7)		

^a^: over all ten modelled seasons

^b^: prediction interval

^c^: number needed to vaccinate

^d^: LAIV-VE was assumed to be 50% higher among 2-6 year old children compared to inactivated vaccines

### Vaccination scenario effectiveness

**Fig. 4:**
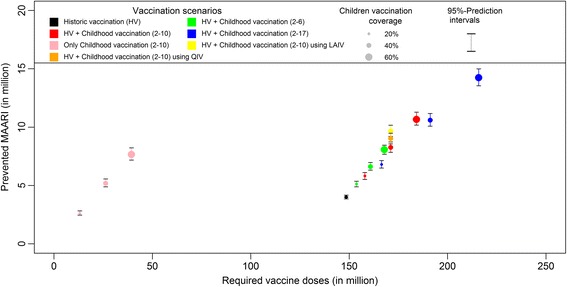
Prevented I-MAARI and required doses for different vaccination strategies. Model-predicted number of prevented I-MAARI and required vaccine doses over ten seasons for each investigated vaccination scenario. Each vaccination scenario is compared to a hypothetical scenario without any influenza vaccination. The scenario “historic vaccination” represents the actual vaccination uptake as estimated for the modelled seasons

**Fig. 5:**
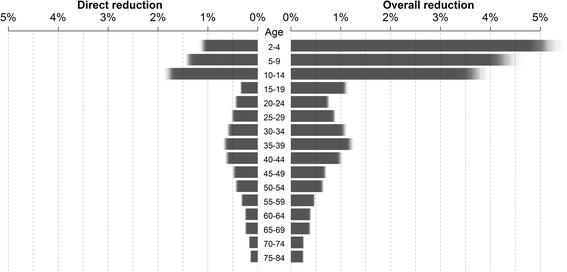
I-MAARI reduction for different allocations of one million vaccine doses. Legend: Predicted direct (left) and overall (right) relative reduction of I-MAARI when allocating one million vaccine doses per year to one single age group (with all other age groups remaining unvaccinated). The respective vaccination target age groups are provided in the middle column

The additional implementation of childhood vaccination yields large indirect effects within the whole population, e.g. reducing I-MAARI among elderly by an additional 11% compared to the historic vaccination scenario (Fig. 3). As a consequence childhood vaccination scenarios always reduced the number needed to vaccinate to prevent one I-MAARI (Table 2). Thus, the historic vaccination scenario is least efficient at preventing I-MAARI as it requires the most vaccine doses to prevent one I-MAARI (Fig. 4). A vaccine doses allocation analyses yields that — under a restriction of only one million available vaccinations per year — the largest overall I-MAARI reduction can be achieved by targeting the age group 2-4 years (5% reduction) whereas targeting any one age group over 60 years reduced I-MAARI by less than 0.4% (Fig. 5).

### Sensitivity analyses

Investigating different modelling scenarios, the results were found to be comparable for models assuming a different contact pattern or no B-lineage cross protection (Table 3). The strongest influence on the model had the assumption of mass-action-transmission, which resulted in a 55.6% relative reduction of I-MAARI.

**Table 3:** Relative impact of additional childhood vaccination (2-10 years; 40% coverage) compared to no vaccination for different model scenarios together with marginal data likelihood corresponding to each model version

Model scenario	Predicted relative reduction (with 95%-PI^a^) of I-MAARI due to childhood vaccination	Marginal (log-)likelihood^b^ of the data
Base model	17.8% (17.1 – 18.7%)	-27077.2
S1: No B-lineage crossprotection	15.3% (14.6 – 16.0%)	-27098.2
S2: Mass-action transmission	56.5% (55.3 – 57.5%)	-30253.9
S3: POLYMOD contact structure	19.5% (18.9 – 20.2%)	-26952.3
S4: Direct vaccination effects only	7.9% (7.7 – 8.1%)	-27084.5

^a^: prediction interval

^b^: The marginal loglikelihoods measures a model capability of explaining the data. Differences greater than five indicate a strong preference for the model yielding a higher likelihood

The highest marginal likelihood of the data was given by the base model and the model assuming a contact pattern according to POLYMOD. The mass-action-transmission model yielded a considerably lower marginal likelihood (Table 3 which corresponds to a visibly worse model fit as displayed in Additional file 1: Figure S6.

## Discussion

We developed a dynamic SEIR-type transmission model to reconstruct the seasonal influenza epidemics between the years 2003/04 and 2013/14. Each epidemic season was represented through its estimated number of I-MAARI and its subtype distribution. When examining hypothetical childhood vaccination scenarios, the fitted model predicted considerable reduction in I-MAARI across all age-groups, which go beyond the expected direct effects. The implementation of a phenomenological transmission rate had a major impact on the predicted effects of augmented vaccination scenarios, since mass-action-transmission models suggested even more drastic incidence reductions.

The observed influenza waves are well reflected by the fitted model; in particular the variability in the magnitude of the epidemic seasons and the circulating subtypes (Fig. 1). The model-predicted epidemics do not capture each nuance of the weekly I-MAARI numbers. However, it should be noted that the weekly I-MAARI are already estimated numbers such that the true I-MAARI curve might be less fluctuate than suggested by the data [20]. This applies in particular for the weeks prior to beginning and after the end of each epidemic season. In those weeks the data yields zero I-MAARI whereas the true number is certainly positive. Nevertheless, the advantage of relying on the estimated I-MAARI is that they represent only influenza-attributable medical consultations. Thus – unlike e.g. ILI incidence data – the I-MAARI are better capable to capture the true influenza epidemics, which directly improves the validity of the fitted model.

Applying a partial stratification of the model parameters offers the advantage that all observed variability in influenza epidemics is explained only by transmission aspects for which variation by season and subtype is epidemiologically plausible. On the contrary, transmission factors that likely stay consistent over the years – such as contact patterns or the consultation rate, i.e. the proportion of sick people seeking medical advice – are enforced to do so. Thus, the stratification approach provides robust estimates for such constant quantities, e.g. the consultation rate of children under 5 years was estimated to be 45.3% over all seasons and subtypes (Table 1). In comparison, by modelling each influenza season separately Baguelin et al. obtained heavily diverse ascertainment probabilities, i.e. probabilities for an influenza case leading to notification. For children under 15 years these ranged between 0.1% and 2% depending on season and subtype [16]. This diversity in the estimates is difficult to explain. Moreover, if the ascertainment probabilities were over- or underestimated it also implies that other parameters might be falsely estimated since the unobserved influenza epidemics may be actually higher or lower, respectively. Secondly, our stratified model framework provides distinct estimates for those parameters where heterogeneity is expected, such as the distinct susceptibility profiles with respect to different subtypes and seasons (Fig. 2). These are in line with available data on population immunity against A(H1N1)pdm09 following the pandemic season 2009/10 in the UK, indicating a very high immunity among elderly and low immunity among children [52]. However, in Germany post-pandemic antibody titres against A(H1N1)pdm09 were comparably low among elderly, although in the subsequent season the lowest infection rates were also detected among elderly [53]. This inconsistency might be caused from pre-existing immunity not measurable through cross-reactive antibodies [53], such that our estimated susceptibilities provide a rough assessment of the actual pre-existing immunity. On the other side, model stratification by season in contrast to utilizing a continuous model as done by Vynnycky et al. and others [17, 18, 37] is necessary to obtain additional insight into the potential range of vaccination impact that might vary by season e.g. due to different VEs. Thus, the model predictions suggest that a strong influenza season is associated with a small relative vaccination impact (compare Figs. 1 and 3), presumably because antigenic virus drift may lead to high population susceptibility and vaccine mismatch at the same time. This association was also observed by more realistic network-based models [54].

As a novel approach our model also incorporated contact data from people with ILI symptoms into the transmission process whereas most other models utilize POLYMOD data that have investigated contact patterns of healthy people only [44, 47]. Although the number of school contacts is reduced in the adjusted contact matrix, children still remain the group with the highest contact frequency and even have a higher number of household contacts such that transmission between children and adults is even more pronounced [48]. Childhood vaccination impact was thus even slightly larger compared to a model utilizing solely the POLYMOD data (S3) (Table 3). Also note that the POLYMOD based model was more likely from a statistical standpoint (Table 3). The worse fit of the model incorporating behavioural changes due to illness might originate from the respective contact data being collected in the UK during the pandemic season and thus these data are not necessarily applicable for seasonal influenza transmission in Germany. However, for the eventual selection of our base analysis we preferred a more realistic model accounting for adjusted contact behaviour over a slightly improved model fit.

### Impact of Childhood vaccination

Investigating a childhood vaccination scenario targeting 2-10 year old children at 40% coverage in addition to the observed vaccination uptake in the past, our model predicts an overall reduction in I-MAARI of 17.8% that is mostly caused by reductions among the vaccination target group of children and elderly themselves. Compared to the expected direct effects of 11.0% decrease in I-MAARI, routine childhood vaccination is predicted to causes considerable positive indirect effects. However, throughout all other influenza transmission models the predicted impact of childhood vaccination is much more pronounced [16–19, 37], e.g. Rose et al. estimated a 40% reduction in symptomatic cases through increased vaccination coverage among children and utilization of LAIV, whereas Baguelin et al. suggest a 55% incidence reduction in a scenario with 50% vaccination coverage among 1-16 year old children.

Those deviating results can be explained by the assumption of a phenomenological transmission rate. Our alternative model scenario (S2) employing the mass-action-principle – like all previous influenza models – also predicts a 56.5% reduction in I-MAARI when vaccinating 40% of 2-10 year-old children (Table 3). In the case of phenomenological transmission (base model) the indirect vaccination effects are much less pronounced, because small reductions in the prevalence of infection have an even smaller effect on the resulting force of infection, whereas when assuming mass-action-transmission these effects are proportional. From a statistical perspective, the phenomenological model is more valid as indicated by its higher marginal likelihood of the data compared to the mass-action-transmission model, which yields a considerably worse fit especially for weak waves such as those in the seasons 2007/08 or 2010/11 (see Additional file 1: Figure S5). Furthermore, the phenomenological modelling approach has a transmission-dynamic justification, since influenza prevalence is often spatially clustered across Germany [21]. This disagrees with the mass-action-principle assuming a homogeneous distribution of infectious people in the population.

Expanding the vaccination program to 11 to 17 year old children leads to an additional 5.1% decrease in I-MAARI (Table 2), whereas increasing the vaccination uptake among children aged 2 to 10 years to 60% leads to an additional 5.2% reduction. Thus, allocating resources to the age-group with the highest contact rate is worth considering. Analogously, administration of a vaccine providing higher VE among children — such as LAIV in our model example — also enhances the vaccination impact. Again note, that the assumption of LAIV granting improved protection must be challenged in the face of new evidence from the US [15]. An additional benefit would also have been provided by quadrivalent vaccines, since in seasons 2005/06, 2007/08 and 2008/09 we observed a considerable circulation of the B-lineage that was missing in the vaccine. Due to indirect effects routine childhood influenza vaccination can prevent more I-MAARI compared to a vaccination strategy among elderly even with 100% uptake. When taking required vaccine doses into account (Fig. 4), childhood vaccination is much more efficient compared to targeted vaccination of high-risk groups as it yields much lower NNVs to prevent one I-MAARI. This results primarily from indirect effects affecting the overall population, which are most pronounced when targeting younger age groups (Figs. 3, 5).

### Limitations

Although the model fit to the I-MAARI and virological data is overall satisfactory, there is one model mismatch in 2003/04 where the virological data detected A(H3N2) as the only circulating subtype whereas the model additionally predicts circulation of B-Yamagata. This is presumably a result of the I-MAARI data forming a double peak which was interpreted as two overlapping waves by the model. In fact, this double peak might have originated from a reduced mid-season transmission activity during school vacation in February 2004, which cannot be captured by the model, or maybe from inaccuracies in the estimation of the I-MAARI.

Although our stratification approach allows for the necessary seasonal heterogeneity when modelling influenza transmission, our model is missing some explicit linkage between subsequent seasons, e.g. acquired immunity could be carried over into the following seasons as implemented by *Goeyvaerts et al.* [36].

We modelled vaccine administration being completed with beginning of each season, which does not account for possible late administration, e.g. in February or March, or for the delay in developing vaccine-induced immunity. This approach thus slightly overestimates the impact of vaccination in our model. However, in the past most vaccines were in fact administered much prior to the beginning of influenza season such that we found this model simplification to have marginal effects [24].

We assumed a fixed probability of 0.67 for developing symptoms for all age groups and subtypes although this probability likely varies accordingly. However, Carrat et al. [49] did not detect any significant differences at least with respect to subtype and estimating this probability as an age and subtype-stratified model parameter would have led to identifiability problems since it functions in a similar way as especially the probability for seeking medical care.

Finally, the focus of our model predictions was on the number of I-MAARI by age groups. Thus, our predictions do not account for different hospitalization or mortality rates which could provide additional insight especially when comparing the overall benefits of vaccination programs targeting different age groups. Moreover, our model did not explicitly account for individuals with an underlying disease, that might suffer from more severe influenza and among which the vaccine might be less effective. However, the highest proportion of influenza-associated complications and mortality is usually seen in young children and the elderly, the same age-groups where our model also predicted the strongest relative reduction in I-MAARI (Fig. 3).

## Conclusion

The present study provides an expansion of existing modelling studies investigating the impact of vaccinating the main spreaders of influenza – children. We employed a stratified model approach that simultaneously addressed seasonal variability but also consistent patterns within influenza transmission dynamics. Among the revisited methodological approaches, we found that a switch from the established mass-action-principle to a phenomenological approach has considerable impact on the predicted effects of childhood vaccination programs. Thus, more insight on the accurate modelling of transmission rates in compartment models or, alternatively, the application of more realistic individual-based models as in Eichner et al. are required in the future [35]. Even with our more conservative approach, our model predicted that childhood vaccination could considerably reduce influenza infections on the ambulant level with a much lower number-needed-to-vaccinate than the currently implemented vaccination strategy.

## Additional file

Additional file 1: Supplementary information. (PDF 2648 kb)

## Abbreviations

AGI: German working group of influenza
MAARI: medically attended acute respiratory infections
I-MAARI: influenza attributable MAARI
TIV: trivalent influenza vaccine
QIV: quadrivalent influenza vaccine
LAIV: live attenuated influenza vaccine
WHO: World Health Organization
I-MOVE: Influenza - Monitoring Vaccine Effectiveness
VE: vaccine effectiveness
NNV: number needed to vaccinate
