# Supplementary material for: Is the impact of childhood influenza vaccination less than expected: a transmission modelling study
Source: BMC Infect Dis. 2017 Apr 11;17:258. doi: 10.1186/s12879-017-2344-6 (PMC5387286; doi:10.1186/s12879-017-2344-6)
Supplement: Additional file 1: — Supplementary information. (PDF 2648 kb) [file 12879_2017_2344_MOESM1_ESM.pdf]

Supplementary Information: Modelling seasonal transmission of influenza in Germany

Felix Weidemann[1], Cornelius Remschmidt[1], Silke Buda[1], Udo Buchholz[1], Bernhard Ultsch[1], and Ole Wichmann*[1]

[1]Department for Infectious Disease Epidemiology, Robert Koch Institute, Berlin, Germany

Aim of this supplementary document is to provide additional details on the mathematical transmission model developed for the work on seasonal influenza presented in the main article, the utilized data and statistical methodology. Moreover, we present some additional results regarding sensitivity analyses. The structure of this document is given through the following table of contents.

Contents

1 Epidemiological Data **3**

1.1 Data on seasonal influenza disease burden . . . 3

1.1.1 Syndromic surveillance: Influenza attributable excess MAARI . . . 3

1.1.2 Virological surveillance . . . 3

1.2 Demographic data . . . 5

1.3 Vaccination coverage . . . 6

1.4 Vaccine effectiveness data . . . 7

1.5 Contact frequency data . . . 8

1.5.1 Contact matrix: healthy people . . . 9

1.5.2 Contact matrix: sick people . . . 10

2 Influenza transmission model **11**

2.1 Basic transmission model structure . . . 11

2.1.1 Initial conditions of the ODE system . . . 12

2.2 Disease transmission . . . 13

*Correspondence to: wichmanno@rki.de

2.2.1 Transmission rate 13
2.2.2 Adjustment of seasonal shift 15
2.3 Number of cases and I-MAARI 16
2.4 Seasonal stratification and model parameters 18

3 Bayesian inference procedure **20**
3.1 Prior elicitation 20
3.2 Likelihood function 21
3.3 Posterior sampling 23
3.4 Inference results 25

4 Sensitivity analyses **25**
4.1 Model version: mass-action-transmission 25
4.2 Model version: disabled indirect effects 28
4.3 Model version: POLYMOD contact matrix 29
4.4 Model version: no B-lineage cross protection 29

1 Epidemiological Data

1.1 Data on seasonal influenza disease burden

The seasonal influenza epidemics were captured via two distinct data sets. Firstly, the magnitude of each epidemic season was given through the influenza attributable number of medically attended acute respiratory infections (MAARI), i.e. the number of influenza excess consultations as estimated in the work by an der Heiden et al. [1]. Secondly, the subtype distribution within each season was derived from virological surveillance data from the National Reference Centre for Influenza located at the Robert Koch Institute. Both of these data sources are also displayed in the annual reports of the German working group on influenza (Arbeitsgemeinschaft Influenza, AGI) (e.g. [32]).

1.1.1 Syndromic surveillance: Influenza attributable excess MAARI

The number of influenza attributable excess MAARI, i.e. the I-MAARI, is estimated based on data from the syndromic sentinel surveillance system of the AGI. The syndromic surveillance measures the age-specific MAARI incidence over the course of each season in Germany.

The influenza attributable proportion among all MAARI was then estimated by utilizing a time series modelling approach as it was described in an der Heiden et al. [1]. The influenza-attributable excess MAARI were defined as the difference between the actual MAARI (measured though the sentinel network) and an estimated baseline within each epidemic season.

The resulting estimates thus provided data on the number of medically attended influenza cases $D_{t,a}^{(s)}$ for each season[1] $s \in \{2004, \dots, 2009, 2011, \dots, 2014\}$, week[2] $t \in \{-12, \dots, 30\}$ and age group a. The AGI considers five age groups: 0-4 years of age (y), 5-14 y, 15-34 y, 35-59 y and $\geq$60 y. The I-MAARI data $\boldsymbol{D}$ is shown in Figure S1.

1.1.2 Virological surveillance

Data on subtype distribution was provided by the National Reference Centre for Influenza and is displayed in the annual AGI reports, e.g. [32]. Each season the reference centre examined a set of specimen (submitted from practitioners participating in the sentinel system) and examined whether the specimen was influenza positive and, if so, determined the influenza subtype.

The possible subtypes were the two types of influenza A (i.e. AH1N1 and AH3N2) and influenza B. The AH1N1 type either referred to the pandemic variant AH1N1pdm09 or to the pre-pandemic variant AH1N1prepan for the seasons following and prior to the year 2010, respectively. With beginning of the season 2008/09, data from the reference centre also distinguished between the two lineages of influenza B (Yamagata and Victoria). For the seasons prior to 2008/09 we assessed the distribution of the B-lineages from the seasonal AGI reports.

Nevertheless, we required data on influenza-positive tests for each B-lineage for the epidemic seasons prior to 2008/09. Thus, we assigned each influenza-B positive specimen to one of the two B-lineages with probability equal to the season-specific lineage share. For instance, for the season 2005/06 each specimen positive for

[1] e.g. $s = 2004$ denotes the season 2003/2004, etc.

[2] e.g. $t = 0$ denotes the last week of calendar year leading into the season, $t = 1$ denotes the second to last week of that year, etc.

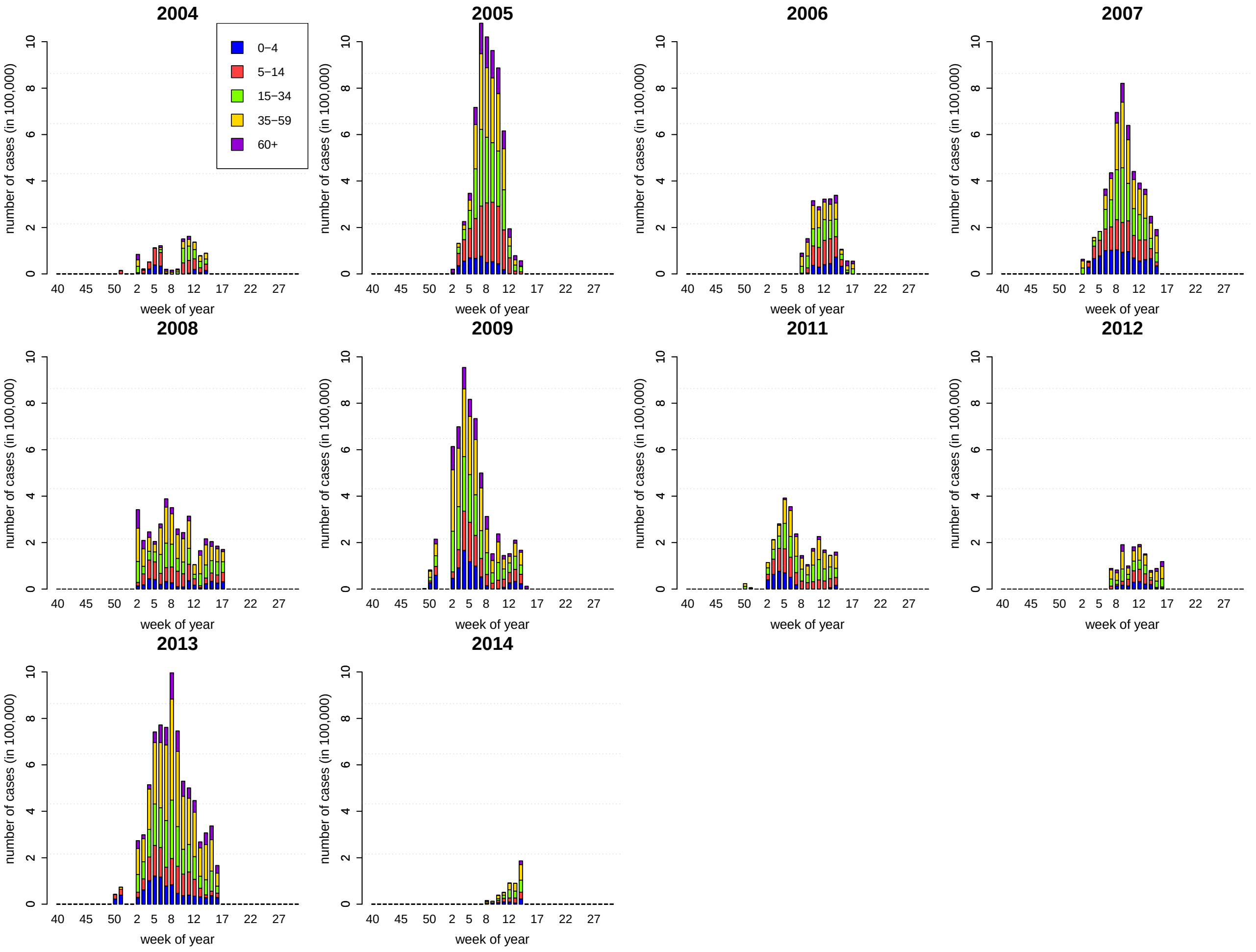

Figure S1: Age-stratified number of weekly I-MAARI for each season from 2003/2004 until 2013/14. (The pandemic season was excluded from the analysis). The year displayed in each subfigure refers to the later half year of the epidemic season.

influenza B was assigned to the B-Victoria lineage with a probability of 90%. Otherwise, it was assigned to the B-Yamagata lineage.

According to the AGI reports, apart from the season 2005/06 there was no other epidemic season with considerable cocirculation of both B-lineages prior to 2009. Thus, the random lineage assignment of the B-positive specimen has only marginal impact on the virological data.

The resulting virological data consisted of numbers on overall influenza positive specimen and its distribution over the four considered subtypes $\boldsymbol{P}_{t,a}^{(s)} = (P_{t,a}^{(s),\text{AH1N1}}, P_{t,a}^{(s),\text{AH3N2}}, P_{t,a}^{(s),\text{B-Vic}}, P_{t,a}^{(s),\text{B-Yam}})$ (i.e. the two A types and the two B-lineages), stratified by seasons $s \in \{2004, \ldots, 2009, 2011, \ldots, 2014\}$, week $t \in \{-12, \ldots, 30\}$ and seven age groups a. These seven age groups were 0-1 y, 2-4 y, 5-14 y, 15-34 y, 35-49 y, 50-59 y and $\geq$60 y. The virological data $\boldsymbol{P}$ (pooled over all age groups) is displayed in Figure S2.

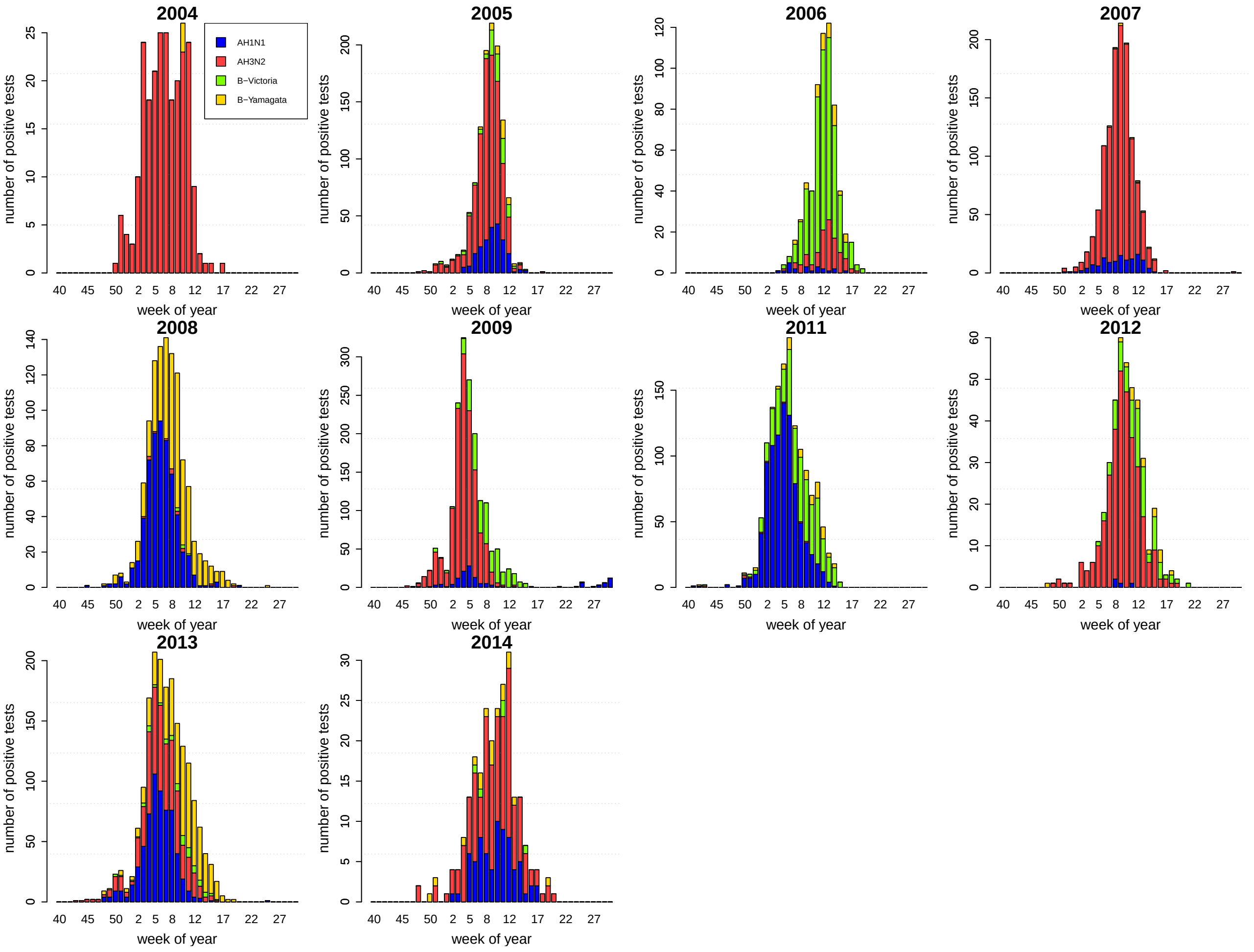

Figure S2: Subtype-specific weekly number of influenza positive specimen as provided by the National Reference Centre for each season from 2003/2004 until 2013/14. (The pandemic season was excluded from the analysis). The year displayed in the title of each subfigure refers to the second half year of the season.

1.2 Demographic data

Data on population counts with respect to yearly age groups was obtained from the Federal Bureau of Statistics [11]. This data covered all years from 2000 up to 2012. To generate population count data for the years 2013 and 2014 we extrapolated the yearly birth cohorts, i.e. the year-specific number of people younger than one year of age. Then we extrapolated the size of existing one-year wide age cohorts into the years 2013 and 2014, subject to the observed survival rates and possible immigration rates.

For instance, to estimate the number of 10 year old children in 2013 we first estimated the rate of change between the population sizes of 9 year old children and 10 year old children for all years from 2001 to 2012. These change rates were then extrapolated to the year 2013 using a linear model. This extrapolated change rate was then applied to the cohort of 9 year old children in 2012 to obtain an estimate for the number of 10 year old children in 2013. This procedure was performed for all one-year wide age cohorts from 1 to 100 years of age and subsequently for both years 2013 and 2014. The resulting population counts as they were applied in the model are displayed in Table S1.

Table S1: Season-specific data/estimates on population counts for five-year age bands in Germany.

Seasonal population counts (in 1,000)										
Age group	Season									
	03/04	04/05	05/06	06/07	07/08	08/09	10/11	11/12	12/13	13/14
0-4	4435	4346	4245	4192	4154	4117	4089	4108	4073	4031
5-9	3984	3972	3963	3896	3797	3714	3575	3529	3503	3490
10-14	4459	4296	4110	4036	4009	3981	3973	3922	3828	3748
15-19	4770	4810	4853	4782	4660	4501	4177	4125	4095	4058
20-24	4919	4930	4886	4881	4876	4927	5066	5044	4934	4775
25-29	4713	4757	4856	4923	4965	4979	4997	5050	5082	5164
30-34	5650	5287	4997	4787	4686	4691	4870	4982	5063	5113
35-39	7111	6928	6678	6389	6020	5599	4986	4813	4744	4779
40-44	6964	7105	7174	7198	7153	7033	6636	6379	6037	5640
45-49	5996	6152	6330	6513	6680	6867	7093	7137	7113	7013
50-54	5496	5554	5593	5676	5761	5875	6213	6408	6587	6785
55-59	4384	4467	4819	5072	5231	5331	5432	5519	5611	5732
60-64	5417	5141	4618	4257	4202	4180	4603	4852	5008	5110
65-69	4882	5110	5292	5378	5242	5064	4318	3981	3937	3927
70-74	3414	3518	3670	3881	4123	4410	4805	4895	4777	4626
75-79	2835	2895	2938	2945	2935	2891	3136	3314	3541	3812
80-84	1909	1965	1985	2001	2028	2097	2191	2209	2179	2137
85-89	720	761	879	998	1088	1138	1199	1225	1266	1315
90-94	353	354	342	323	306	310	386	422	461	496
95-99	73	72	71	72	72	77	82	89	94	101

1.3 Vaccination coverage

Age and seasons specific vaccination coverage rates were based on statutory health insurance claims data which were routinely collected at the Robert Koch Institute [31].

These data contained numbers on mandatorily insured people covered by each federal health insurance, stratified by year of birth and season from 2003 till 2014. Additionally, the data provided the number of monthly administered influenza vaccine doses stratified by age group. Lastly, the data contained numbers on the population sizes within each region of the respective federal health insurances. Some federal insurances started to report the administration data not in 2004, but in a later season instead. Thus, coverage estimates from the early years could not be obtained for each region.

The data allowed to estimate monthly influenza vaccination coverage rates within each season from 2004 till 2014, stratified by yearly birth cohort and region, subject to data availability. By weighting the available region specific estimates with the regions populations sizes, this yielded an overall estimate for the monthly coverage rates in Germany. Since almost all influenza vaccine doses were administered prior to beginning of each season, for each season the monthly coverage rates were pooled to obtain an overall estimate of the influenza vaccination coverage by age group. The so derived coverage estimates for the seasons 2004/2005 till 2013/2014 are displayed in Table S2. For the season 2003/04 we assumed the vaccination coverage to be the same as in 2004/05.

Table S2: Season-specific estimates for influenza vaccination coverage stratified by age groups. Estimates are based on health insurance claims data [31].

Seasonal vaccination coverage (in %)									
Age group	Season								
	04/05	05/06	06/07	07/08	08/09	10/11	11/12	12/13	13/14
0-4	3.4	5.9	4.1	4.1	3.8	3.7	3.4	2.9	3.0
5-9	5.0	8.7	6.5	6.7	6.2	5.9	5.5	4.7	4.7
10-19	5.4	8.0	5.4	5.5	4.8	4.6	4.4	3.9	4.0
20-29	4.9	7.0	4.8	5.0	4.4	3.9	3.6	2.9	3.1
30-39	7.2	9.9	7.3	7.4	6.7	6.1	5.7	4.6	5.0
40-49	11.2	14.5	11.5	11.6	10.6	9.7	9.1	7.6	8.0
50-59	19.4	24.2	20.5	20.3	18.8	16.7	15.7	13.3	13.7
60-69	38.0	45.6	42.3	41.6	39.2	34.5	32.2	28.1	28.6
70-79	48.4	55.5	53.3	53.2	51.4	47.8	46.4	42.4	43.7
80+	51.1	57.7	56.2	56.8	55.7	51.9	50.8	46.6	47.9

1.4 Vaccine effectiveness data

To obtain estimates on influenza vaccine effectiveness (VE) for the seasons 2003/04 until 2013/14, we relied on three different sources: (1) data from the European network *"Influenza - Monitoring Vaccine Effectiveness"* (I-MOVE) providing subtype and age specific estimates for each season beginning in 2008/09 [20, 21, 22, 23, 35, 36], (2) data from Cochrane reviews measuring the influenza vaccine effectiveness for children and adults stratified by strain match of the vaccine [8, 18], and (3) data from the AGI reports providing WHO recommendations on strain components in the vaccine and the observed strain match within each season.

The final transmission model required VE estimates for each season, subtype and age group. Since such detailed data with respect to age was not available we defined three age groups ($<$15y, 15-59y, $\geq$60y) assuming the VE within each of these age groups to be equal within each season. To inform each of the 120 VE estimates (10 seasons, 4 subtypes, 3 age groups) we applied the following hierarchical procedure:

1. For influenza A, if data from I-MOVE was available, we applied the best available estimate to specify the VE for each subtype, season and age group (i.e. adjusted for poss. risk factors and site $>$ adjusted for site only $>$ crude estimate).

2. For well-matched seasons (according to AGI reports) and if no I-MOVE data was available, we applied the mean VE estimates from all I-MOVE studies subject to subtype and age group.

3. For poorly-matched seasons (according to AGI reports) and if no I-MOVE data was available, we applied the VE estimated from well-matched seasons reduced by 10 percentage points, as this was the observed VE difference according to the cochrane reviews [8, 18].

4. Regarding influenza B, the I-MOVE VE estimates were not referring to one B-lineage but to the B-lineage mix instead. Thus, we derived the two B-lineage specific VE estimates taking the observed lineage mix and a B-lineage cross-protection of 60% into account. This cross-protection refers to the vaccine induced protection against the B-lineage that was not included in the trivalent vaccine according to WHO recommendation. A cross-protection of 60% compared to the effectiveness against the B-lineage

that is included in the vaccine was detected in a few clinical studies and has already been used within other transmission models [10, 25, 34]. Thus, for instance in the season 2012/13 I-MOVE estimated the influenza B VE of 44% among elderly. The B-Yamagata lineage had a share of 90% among circulating B-lineages which – in combination with the assumption of 60% crossprotection – thus yielded estimated VEs of 46% and 28% against B-Yamagata and B-Victoria, respectively.

The resulting comprehensive VE matrix is given by Table S3.

Table S3: VE estimates applied in the transmission model. Yellow: estimates based on I-MOVE data. Orange: mean estimates for B-lineage included in vaccine. Red: VE for B-lineage not included in vaccine. Green: mean VE for A strains in well-matched seasons. Violet: mean VE for A strains in poorly-matched seasons.

Year	**VE** (in %) in age group **$<$15**, **15-59** and **$\geq$60**			
	AH1N1	AH3N2	B Yama.	B Vict.
2014	(64, 39, 52)	(30, 7, 47)	(38, 67, 52)	(23, 40, 31)
2013	(36, 56, 59)	(36, 44, 37)	(24, 66, 46)	(23, 40, 31)
2012	(65, 50, 60)	(19, 63, 15)	(23, 40, 31)	(38, 67, 52)
2011	(77, 27, 72)	(38, 44, 39)	(23, 40, 31)	(52, 68, 60)
2010	(85, 73, 78)	(38, 44, 39)	(23, 40, 31)	(38, 67, 52)
2009	(55, 40, 50)	(56, 56, 56)	(38, 67, 52)	(23, 40, 31)
2008	(65, 50, 60)	(38, 44, 39)	(23, 40, 31)	(38, 67, 52)
2007	(55, 40, 50)	(38, 44, 39)	(23, 40, 31)	(38, 67, 52)
2006	(65, 50, 60)	(28, 34, 29)	(38, 67, 52)	(23, 40, 31)
2005	(65, 50, 60)	(28, 34, 29)	(38, 67, 52)	(23, 40, 31)
2004	(65, 50, 60)	(28, 34, 29)	(38, 67, 52)	(23, 40, 31)

1.5 Contact frequency data

Although a successful person-to-person transmission of influenza requires more than a sole social contact – namely a given susceptibility of the affected person and a degree of virus transmissibility – the actual frequency of social contacts play a central role in transmission modelling, especially when considering age-structured models. For informing the contact matrix employed in our here developed transmission model we utilised two different sources of data, i.e.

- Firstly, we applied data from a European wide survey on the frequency on social contact, also known as POLYMOD [28]. Using this data is by now standard when it comes to modelling the spread of airborne diseases and other person-to-person transmitted pathogens.
- Additionally, we incorporated data measuring the impact of illness on social behaviour as it was assessed by Van Kerckhove et al. [37] and Eames et al. [9]. Since influenza is primarily transmitted by symptomatically ill people, the actual contact frequencies leading to transmission are not accurately represented through the POLYMOD data (which assessed the contact behaviour of healthy people). Thus, the POLYMOD contact matrix has to be adjusted to account for the changed contact pattern of sick people.

To derive a contact matrix used within the eventual model, we proceeded in two steps. First, we estimated a contact matrix subject to healthy people using the German part of the POLYMOD data. In a second step, this matrix was processed with the relative age-specific contact frequency changes as measured by Van Kerckhove et al. [37] and Eames et al. [9] to obtain a contact matrix subject to symptomatically ill people. These two steps are outlined in the following.

1.5.1 Contact matrix: healthy people

The POLYMOD survey contains data on the number of daily contacts of each participant, with additional information on the age, duration and quality (e.g. physical vs. non-physical) of each contact. The German part of the survey consisted of filled out contact diaries from 1185 participants.

For our purpose of deriving a contact matrix for transmission of influenza we extracted only physical contacts lasting longer than 15 minutes. The resulting contact data consisted of data points

$$\boldsymbol{c}^{(i)} = \left(c_0^{(i)}, c_1^{(i)}, \ldots, c_{99}^{(i)}, c_{100}^{(i)}\right), \quad i = 1, \ldots, 1185$$

where each component $c_j^{(i)}$ denotes the number of contacts of participant i with people of age j, i.e. $\boldsymbol{c}^{(i)}$ summarizes all contacts of participant i. We utilized these data to estimate an age-structured contact matrix

$$\boldsymbol{\beta} = \left\{\beta_{kj}\right\}_{k,j=0,\ldots,100},$$

where β_{kj} refers to average number of daily contacts from individuals of age k with individuals of age j. The matrix $\boldsymbol{\beta}$ was parametrized using a two dimensional third-order B-spline basis

$$\boldsymbol{B} = (B_1(a), \ldots, B_{18}(a)),$$

defined on 15 knots over the age range $a \in [0, 80]$. Thus, the components of the parametrized contact matrix $\boldsymbol{\beta}(\boldsymbol{\phi})$ are given by

$$\beta_{kj}(\boldsymbol{\phi}) = \sum_{l=1}^{18} \sum_{n=1}^{18} \phi_{l,n} B_l(k) B_n(j), \tag{1}$$

subject to the parameter vector $\boldsymbol{\phi} = \{\phi_{l,n}\}_{l,n=1,\ldots,18}$. The parameter $\boldsymbol{\phi}$ was estimated such that it maximizes the likelihood $L(\boldsymbol{c}|\boldsymbol{\phi})$ of observing the contact data $\boldsymbol{c} = \left\{\boldsymbol{c}^{(i)}\right\}$, i.e.

$$\hat{\boldsymbol{\phi}} = \arg\max_{\boldsymbol{\phi}} L(\boldsymbol{c}|\boldsymbol{\phi}), \tag{2}$$

with

$$L(\boldsymbol{c}|\boldsymbol{\phi}) = \prod_{i=1}^{1185} \prod_{j=0}^{80} \frac{\beta_{a_i j}(\boldsymbol{\phi})^{c_j^{(i)}}}{c_j^{(i)}!} e^{-\beta_{a_i j}(\boldsymbol{\phi})}, \tag{3}$$

where the likelihood function L implies that the contact frequencies $c_j^{(i)}$ are assumed to be Poisson-distributed with expectation $\beta_{a_i j}(\boldsymbol{\phi})$, where a_i denotes the age of participant i. Alternative approaches for a spline-based estimation of the contact matrix utilizing the POLYMOD data can be found in e.g. Goeyvaerts et al. [15]. The fitted matrix is displayed in Figure S3 (left figure).

1.5.2 Contact matrix: sick people

Data on changes in the contact behaviour caused by symptomatic illness was collected by Eames et al. [9] and previously analysed by Eames et al. [9] and Van Kerckhove et al. [37].

Here we utilized results from Van Kerckhove et al. [37] who estimated a pair of physical contact matrices based on diaries from symptomatic individuals and from the same individuals after recovery from illness, respectively. These two matrices contain contact rates $\boldsymbol{C}^{(\text{healthy})} = \left\{C_{kj}^{(\text{healthy})}\right\}$ (and analogously $\boldsymbol{C}^{(\text{sick})}$) stratified by six different age groups ($k, j = 1, \ldots, 6$), i.e. 0-3 y, 4-10 y, 11-21 y, 22-45 y, 46-64 y, and 65-80 y. Thus, $C_{kj}^{(\text{healthy})}$ provides the average daily number of physical contacts of a healthy individual from age group k with any people of age group j, whereas $C_{kj}^{(\text{sick})}$ provides the corresponding number of physical contacts from sick people. The necessary data was provided by the authors upon request.

As the contact matrices contained some zero entries which complicated the comparison, we pursued a Bayesian approach to adjust the contact rates in the data. For that, we assumed each contact rate is a priori $\Gamma(2,2)$-distributed (i.e. has mean one), whereas the measured contact frequencies in the data was assumed to be Poisson-distributed with mean $C_{kj}^{(\text{healthy})}$ or $C_{kj}^{(\text{sick})}$, respectively. Accounting for the number n_k of survey participants within each of the six considerd age groups, this leads to adjusted posterior mean contact frequencies $\widehat{C}_{kj}^{(\bullet)}$, i.e.

$$\widehat{C}_{kj}^{(\bullet)} = \frac{1 + n_i C_{kj}^{(\bullet)}}{2 + n_i}, \qquad \bullet \in \{\text{healthy, sick}\}, \tag{4}$$

such that the adjusted contact frequencies were strictly positive. Based on $\widehat{C}_{kj}^{(\bullet)}$ ($k, j = 1 \ldots, 6$, $\bullet \in \{\text{healthy, sick}\}$) we constructed a contact frequency matrix $\left\{\hat{c}_{kj}^{(\bullet)}\right\}_{k,j=0,\ldots,80}$ employing one-year age groups up to 80 years of age, by equally distributing the estimated average number of contacts over all one-year age groups. From that we calculated the relative age-specific change in contact frequency due to symptomatic sickness $\left\{\hat{r}_{kj}\right\}_{k,j=0,\ldots,80}$, i.e.

$$\hat{r}_{kj} = \frac{\hat{c}_{kj}^{(\text{sick})}}{\hat{c}_{kj}^{(\text{healthy})}}, \qquad k, j = 0, \ldots, 80, \tag{5}$$

which gives the relative reduction (or increase) in the frequency of physical contacts from individuals of age k with individuals of age j.

By construction the resulting matrix $\left\{\hat{r}_{kj}\right\}$ was piecewise constant along the six age groups defined by Van Kerckhove et al. [37]. To obtain a smoothed matrix, we again applied a B-Spline approximation which yields a parametrized matrix $\left\{r_{kj}(\psi)\right\}$. The spline coefficients ψ were estimated by minimizing the squared error sum, i.e.

$$\hat{\psi} = \arg\min_{\psi} \sum_{k=0}^{80} \sum_{j=0}^{80} \left(r_{kj}(\psi) - \hat{r}_{kj}\right)^2. \tag{6}$$

Thus, $\left\{r_{kj}(\hat{\psi})\right\}$ provides the contact adjustment matrix due to symptomatic illness based on the data by Van Kerckhove et al. [37] and Eames et al. [9]. This matrix was then applied to the contact behaviour as derived for healthy people in the proceeding section, which yields a final contact frequency matrix $\boldsymbol{\beta}^{(\text{sick})}$ to be applicable for sick individuals, i.e.

$$\beta_{kj}^{(\text{sick})} = \beta_{kj}(\hat{\phi}) \cdot r_{kj}(\hat{\psi}). \tag{7}$$

This matrix is also displayed in Figure S3 (right figure).

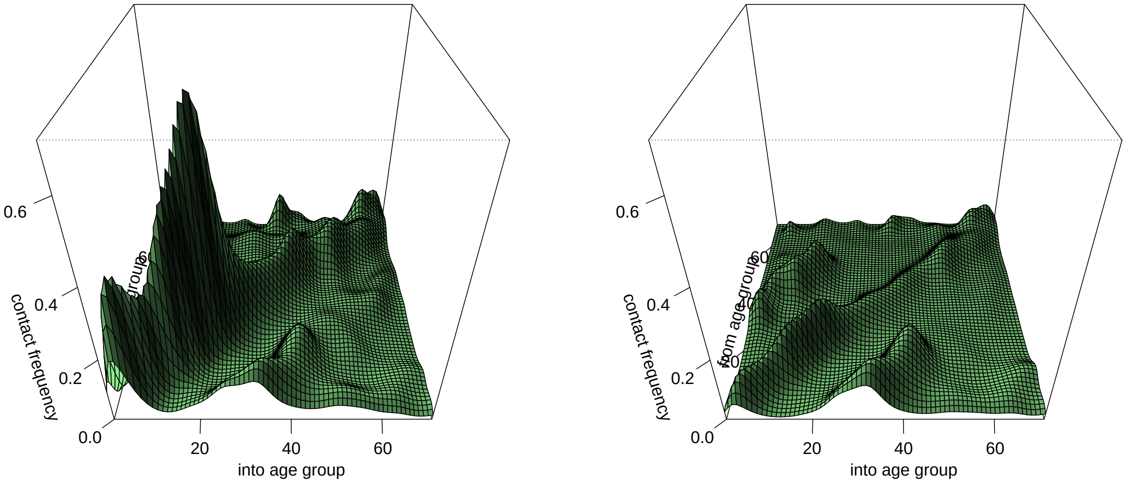

Figure S3: Estimated contact matrices providing the age-specific contact frequencies of healthy (left) and symptomatically ill (right) people.

2 Influenza transmission model

The dynamic transmission of influenza was captured by a mathematical model governing several levels from pathogen transmission to developing symptoms to assessing the associated number of influenza attributable MAARI and subtype distribution. The unobserved disease transmission process within the population was modelled through a system of ordinary differential equations. The corresponding number of influenza cases, excess I-MAARI, and subtype distribution were then derived from the solution of the ODE system and thus provided the link from the hidden transmission dynamics to the observed data. In this section, we will explain the details (and provide corresponding equations) of each aspect from the underlying transmission model and observational component. Details on the model inference procedure will be presented in Section 3.

The overall model time horizon is given through the 10 epidemic seasons from 2003/04 to 2013/14 excluding the pandemic season 2009/10.

2.1 Basic transmission model structure

To capture the hidden transmission dynamics within the German population for each influenza season we implemented a deterministic SEIR-type model, as we were inspired by the models from Baguelin et al. [3], Goeyvaerts et al. [16], Meeyai et al. [27], Pitman et al. [29], Rose et al. [33], Vynnycky et al. [38]. These

dynamics are represented by the following system of ordinary differential equations.

$$
\begin{aligned}
\frac{dS_i}{dt} &= -\lambda_i(t,\boldsymbol{I}(t),\boldsymbol{I}^V(t))S_i & \frac{dS_i^V}{dt} &= -\lambda_i(t,\boldsymbol{I}(t),\boldsymbol{I}^V(t))S_i^V \\
\frac{dE_i}{dt} &= \lambda_i(t,\boldsymbol{I}(t),\boldsymbol{I}^V(t))S_i - \gamma E_i & \frac{dE_i^V}{dt} &= \lambda_i(t,\boldsymbol{I}(t),\boldsymbol{I}^V(t))S_i^V - \gamma E_i^V \\
\frac{dI_i}{dt} &= \gamma E_i - \gamma I_i & \frac{dI_i^V}{dt} &= \gamma E_i^V - \gamma I_i^V \\
\frac{dR_i}{dt} &= \gamma R_i & \frac{dR_i^V}{dt} &= \gamma R_i^V
\end{aligned} \tag{8}
$$

The variables S_i, E_i, I_i, R_i provide the absolute numbers of susceptibles, latently infected, infectious, and immune people, stratified by n_A age groups (index $i = 1\ldots,n_A$). The variables $S_i^V, E_i^V, I_i^V, R_i^V$ provide the corresponding number of vaccinated individuals within the four considered states. We defined overall $n_A = 21$ disjoint age groups, where the respective upper age bounds are given through the vector (0,1,2,4,7,10,14,19,24,29, 34,39,44,49,54,59,64,69,79,89,100).

The term $\lambda_i(t,\boldsymbol{I},\boldsymbol{I}^V)$ represents the force of infection affecting age group i, which is a function that depends among other parameters on the age-stratified number of infected people $\boldsymbol{I}(t)$ and $\boldsymbol{I}^V(t)$. (Notation: $\boldsymbol{I} = (I_1,\ldots,I_{n_A})$)

In contrast to the models presented in Goeyvaerts et al. [16], Pitman et al. [29], Rose et al. [33], Vynnycky et al. [38], the here presented transmission model was applied for each season and each of the four considered influenza subtypes separately. Such a separation approach was also pursued by Baguelin et al. [3] and Meeyai et al. [27]. Hence, interseasonal dynamics (e.g. acquired immunity that lasts more than one season) or cross protection between the subtypes were not *directly* implemented into the model.

The time horizon of each season was $t \in [-17,25]$, where one time unit represents on week. That means each model season began at calender week 35 of the preceding calender year ($t = -17$) and continued over the winter until calender week 25, i.e. from September till June.

This model did not account for any demographic dynamics in the form of birth and death rates, since we assumed that minor demographic developments could be neglected for the short seasonal model time horizon of 42 weeks. Constant population sizes were also considered in the seasonal model by Baguelin et al. [3]. However, the model population counts differed from season to season subject to data from the Federal Statistical Office (Section 1.2).

2.1.1 Initial conditions of the ODE system

In each season and for each subtype individuals either started as susceptible (S) or immune (R) at the beginning of each season. Infection was initially introduced into the model population through a constant outside force of infection affecting every susceptible individual as it will be later described in Section 2.2.

Which fraction of the population starts as susceptible vs. immune and vaccinated vs. unvaccinated at the beginning of each model season depends on two factors: (i) an already existing subtype and age specific immunity and (ii) the season specific vaccination coverage and effectiveness.

Regarding the pre-existing age-specific susceptibility profile we differentiated between the four subtypes AH1N1, AH3N2, B-Yamagata and B-Victoria and additionally distinguished between the pre-pandemic and post-pandemic

AH1N1 strain (AH1N1prepan vs. AH1N1pdm09). For each subtype we considered the same three age bands (<15 y, 15-59 y, ≥60 y) to define age specific susceptibilities $\sigma_a^{(z)}$, i.e.

$$\boldsymbol{\sigma} = \left\{\sigma_a^{(z)}\right\}, \quad a \in \{< 15\text{y}, 15\text{-}59\text{y}, \geq 60\text{y}\}, \quad z \in \{\text{AH1N1prepan}, \text{AH1N1pdm}, \text{AH3N2}, \text{B-Yam}, \text{B-Vic}\}.$$

Since these 15 parameters represent a pre-existing immunity in the population, originating from the circulation of the given subtypes in earlier decades, we assumed this susceptibility within the youngest age band to be complete, i.e. $\sigma_{<15\text{y}}^{(z)} = 1$ for all subtypes z.

In order to additionally consider immunity induced from shortly past influenza seasons, we introduced parameters $\varphi^{(s,z)}$ that control which fraction of the non-immune population (with respect to subtype z) is in fact susceptible for a given season s.

The vaccine coverage $\text{VC}_i^{(s)} \in [0,1]$ and effectiveness $\text{VE}_i^{(s,z)} \in [0,1]$ as given in Sections 1.3 and 1.4 also depend on either age group i and season (s) or on age group i, season (s) and subtype (z), respectively. Hence, we defined the following initial model state at time $t_0 = -17$ for a certain subtype season (s,z) as given below. For easier readability, we dropped the upper index (s,z) from the state variables $(S(t), E(t), \dots)$ although the model is run separately for each season and subtype.

$$\begin{aligned}
S_i(t_0) &= \varphi^{(s,z)}\sigma_{a_i}^{(z)}(1-\text{VC}_i^{(s)})N_i & S_i^V(t_0) &= \varphi^{(s,z)}\sigma_{a_i}^{(z)}(1-\text{VE}_i^{(s,z)})\,\text{VC}_i^{(s)}\,N_i \\
E_i(t_0) &= 0 & E_i^V(t_0) &= 0 \\
I_i(t_0) &= 0 & I_i^V(t_0) &= 0 \\
R_i(t_0) &= (1-\varphi^{(s,z)}\sigma_{a_i}^{(z)})(1-\text{VC}_i^{(s)})N_i & R_i^V(t_0) &= \left(1-\varphi^{(s,z)}\sigma_{a_i}^{(z)}(1-\text{VE}_i^{(s,z)})\right)\text{VC}_i^{(s)}\,N_i
\end{aligned} \tag{9}$$

Here, N_i denotes the size of age group i at the beginning of season s and $a_i \in \{< 15\text{y}, 15\text{-}59\text{y}, \geq 60\text{y}\}$ refers to the age band in which age group i is included. Note, that the age groups $i = 1, \dots, n_A$ considered in the model are a finer decomposition of the age groups for stratification of susceptibility or data.

2.2 Disease transmission

This section provides a full description of the mathematical implementation of the disease transmission rate, i.e. the force of infection, within the model. This includes aspects regarding the age dependent contact frequencies, seasonality, transmissibility, and potential spatial clustering.

2.2.1 Transmission rate

The transmission rate $\lambda_i(t, \boldsymbol{I}, \boldsymbol{I}^V)$ measures the per person force of infection and thus controls the rate at which available susceptibles will become infected within a time unit (i.e. one week in our case) subject to the ODE system given above. The full functional term of the force of infection is defined by

$$\boldsymbol{\lambda}(t, \boldsymbol{I}, \boldsymbol{I}^V) = R_e\gamma \exp\left\{\delta \sin\left(2\pi\left(\frac{t}{52} - t_z + t_s\right)\right)\right\}\boldsymbol{\beta}^{(\text{eff})}\left(\frac{\boldsymbol{I}+\boldsymbol{I}^V}{\boldsymbol{N}}\right)^{\rho} + \lambda_o, \tag{10}$$

which gives the vector of age specific force of infections $\lambda_i(t, \boldsymbol{I}, \boldsymbol{I}^V)$. This term includes several quantities affecting the time specific transmission rate, which will be explained in the following.

Firstly, it contains two constants, the baseline transmissibility R_e and the recovery rate γ. The transmissibility parameter R_e can be interpreted as a measure for the effective reproduction rate (although it does not coincide with the effective reproduction rate), since it directly scales the overall force of infection. The recovery rate γ is included in the functional term in order to avoid parameter collinearity between the recovery rate and R_e, since the time and age specific reproduction rate is given through $\lambda_i(t, \boldsymbol{I}, \boldsymbol{I}^V)/\gamma$, which is then unaffected by the recovery rate and primarily controlled by R_e.

Secondly, the force of infection contains a purely time-dependent term $\exp(\delta \sin(2\pi(t/52 - t_z + t_s)))$ subject to the parameters δ, t_z and t_s. This term entails the within season variation of the transmission rate due to external (e.g. environmental) changes. In that regard, the parameter δ controls the magnitude of the within-season variation whereas the time points t_s and t_z determine the peak transmission time, i.e. the time in the season yielding the highest transmissibility, which might vary with respect to season s and subtype $z \in \{\text{AH1N1}, \text{AH3N2}, \text{B-Yam}, \text{B-Vic}\}$ (not distinguishing between pre-pandemic and pandemic AH1N1).

The third component of the transmission rate is the effective contact matrix $\boldsymbol{\beta}^{(\text{eff})}$, which provides the link between susceptible individuals and the infectious fraction of the population. The effective contact matrix is a composition of the two contact matrices subject to healthy individuals and symptomatically ill individuals, i.e. $\boldsymbol{\beta}^{(\text{healthy})}$ and $\boldsymbol{\beta}^{(\text{sick})}$ according to Section 1.5, respectively. Thus, we define

$$\boldsymbol{\beta}^{(\text{eff})} = \nu(m)^{-1} \left(\boldsymbol{\beta}^{(\text{healthy})} + m\boldsymbol{\beta}^{(\text{sick})} \right), \tag{11}$$

as also suggested by Van Kerckhove et al. [37], where the mixing parameter m controls the relative share of the illness-adjusted contact matrix. This relative share m summarizes two aspects: (i) what proportion of infectious people actually develop symptoms and thus change their contact behaviour according to $\boldsymbol{\beta}^{(\text{sick})}$, and (ii) by how much symptomatically ill individuals are more infectious compared to asymptomatically infected individuals.

Furthermore, the effective contact matrix is normalized by the factor $\nu(m)$ which we defined as the maximum eigenvalue of $\boldsymbol{\beta}^{(\text{healthy})} + m\boldsymbol{\beta}^{(\text{sick})}$ adjusted for the susceptible fractions $\boldsymbol{\sigma}$ within the population. Thus, by writing the age, season and subtype specific susceptible fractions $\left\{\varphi^{(s,z)}\sigma_i^{(z)}\right\}$ as a diagonal matrix $\Sigma = \varphi^{(s,z)} \cdot \text{diag}(\sigma_{a_1}^{(z)}, \ldots, \sigma_{a_{n_a}}^{(z)})$ we computed the maximum eigenvalue of the next-generation-matrix $\Sigma \cdot (\boldsymbol{\beta}^{(\text{healthy})} + m\boldsymbol{\beta}^{(\text{sick})})$. Since this eigenvalue, i.e. $\nu(m)$, is commonly interpreted as a measure for the reproduction rate ([2]), a normalization of the matrix using $\nu(m)$ implies that within our employed model the effective reproduction number is primarily controlled by the parameter R_e. See e.g. Birrell et al. [5] for a similar approach on normalization of the contact matrix.

The final (but most causal) multiplicative component of the transmission rate is a measure for the momentaneous infectious fraction within the population stratified by age. Whereas most influenza transmission models assume the standard mass-action-principle – which implies that the force of infection is proportional to the infectious fraction – here we implemented a so-called phenomenological transmission rate [26], i.e. the force of infection is a concave function with respect to the infectious fraction:

$$\boldsymbol{\lambda}(t, \boldsymbol{I}, \boldsymbol{I}^V) \propto \left(\frac{\boldsymbol{I} + \boldsymbol{I}^V}{\boldsymbol{N}} \right)^{\rho}, \tag{12}$$

where $\boldsymbol{I}$ and $\boldsymbol{I}^V$ provide the age stratified number of infected people and $\boldsymbol{N}$ is the vector of age group sizes. The reasoning for this modelling is that an infection is commonly spatially clustered within a population such that already infected people are more likely to have contact to other infected individuals as compared to yet uninfected people. This also implies that primary infected people have more transmission-relevant contacts

than secondary infected cases. This phenomenon is mathematically reflected by the power parameter ρ, which controls the additional marginal force of infection caused by an increasing infectious fraction. The relevance of non-linear transmission rates was also discussed by Chowell et al. [7] and Kong et al. [24] and a phenomenological transmission approach was already applied for measles transmission by Finkenstdt and Grenfell [12].

The last term within the transmission rate – an additive term – is the already mentioned outside force of infection λ_o which provides a constant chance that susceptible people become infected from an external (from outside of the German population) source of infection, for instance due to abroad stays. This is the initial cause of an influenza epidemic in our model, since there are no initially infectious people within the population at beginning of each model season.

Breaking down the force of infection $\boldsymbol{\lambda}(t,\boldsymbol{I},\boldsymbol{I}^V)$ into its a age specific components $\lambda_i(t,\boldsymbol{I},\boldsymbol{I}^V)$ for $i=1,\ldots,n_a$ we obtain

$$\lambda_i(t,\boldsymbol{I},\boldsymbol{I}^V) = R_e\gamma\exp\left\{\delta\sin\left(2\pi\left(\frac{t}{52}-t_z+t_s\right)\right)\right\}\sum_{j=1}^{n_a}\beta_{ji}^{(\text{eff})}\left(\frac{I_j+I_j^V}{N_j}\right)^{\rho}+\lambda_o. \tag{13}$$

2.2.2 Adjustment of seasonal shift

Considering the transmission rate $\boldsymbol{\lambda}(t,\boldsymbol{I},\boldsymbol{I}^V)$ as defined above, it creates an issue of parameter collinearity regarding the seasonality in transmission. We would like the timing of the influenza seasonality to be primarily controlled by the parameters t_z and t_s within the sinus function in the seasonal transmission rate. However, variations in the parameters R_e, δ, ρ and φ also affect the starting time of the seasonal wave, since these parameters also influence at which time point an infectious fraction $(\boldsymbol{I}+\boldsymbol{I}^V)/\boldsymbol{N}$ is capable to reproduce itself subject to $\boldsymbol{\lambda}(t,\boldsymbol{I},\boldsymbol{I}^V)$.

To dampen this effect, we applied an additive adjustment of the parameter t_z, i.e. $\tilde{t}_z(R_e,\delta,\rho,\varphi)=t_z+c(R_e,\delta,\rho,\varphi)$ such that the influence on seasonality from the parameters R_e, δ, ρ, and φ are roughly neutralized by the additive shift term.

However, note that the interactions between the variables are complex and thus the seasonal adjustment procedure suggested here is very crude: The underlying idea is that there exists an infectious fraction $\boldsymbol{I}^*=f\boldsymbol{N}$ which is capable of reproducing itself, i.e. it fulfils the condition

$$\boldsymbol{I}^* = \frac{\boldsymbol{\lambda}(t,\boldsymbol{I}^*)}{\gamma} = R_e\exp\left\{\delta\sin\left(2\pi\left(\frac{t}{52}-t_z+t_s\right)\right)\right\}\|\varphi\boldsymbol{\sigma}\boldsymbol{\beta}^{(\text{eff})}\|\left(\frac{\boldsymbol{I}^*}{\boldsymbol{N}}\right)^{\rho}. \tag{14}$$

For this illustration we did not distinguish between vaccinated and non-vaccinated individuals and we also omitted the outside force of infection, since we believe that this term is negligible once infection can reproduce itself. Here, we also think of $\boldsymbol{I}^*$ as the total number of infectious people in the population (rather than a vector of age stratified numbers) such that we switched from a matrix equation to a scalar equation and substituted $\boldsymbol{\beta}^{(\text{eff})}$ by $\|\varphi\boldsymbol{\sigma}\boldsymbol{\beta}^{(\text{eff})}\|$, which gives the mean row sums of $\boldsymbol{\beta}^{(\text{eff})}$ while also accounting for existing immunities $\varphi\boldsymbol{\sigma}$. Thus, $\|\varphi\boldsymbol{\sigma}\boldsymbol{\beta}^{(\text{eff})}\|(\boldsymbol{I}^*/\boldsymbol{N})^{\rho}$ gives the mean (with respect to age group) force of infection caused by $\boldsymbol{I}^*$. Substituting the fraction $\boldsymbol{I}^*=f\boldsymbol{N}$ then yields the condition

$$f = R_e\exp\left\{\delta\sin\left(2\pi\left(\frac{t}{52}-t_z+t_s\right)\right)\right\}\|\varphi\boldsymbol{\sigma}\boldsymbol{\beta}^{(\text{eff})}\|f^{\rho}. \tag{15}$$

We are now interested in the time point t^* that fulfils the condition, which is the time point upon which the infections starts to (over)reproduce itself (due to the sinusoidal shape). This solution is given by

$$\frac{t*}{52} = t_z+t_s+\frac{1}{2\pi}\arcsin\left(\delta^{-1}\left((1-\rho)\log f-\log(R_e\|\varphi\boldsymbol{\sigma}\boldsymbol{\beta}^{(\text{eff})}\|\right)\right). \tag{16}$$

As presumed, the solution t^* obviously not only depends on f, t_z and t_s, but also on other parameters. Therefore, the adjustment term $c(R_e, \delta, \rho, \varphi)$ is defined such that the associated solution t^* is roughly unaffected by R_e, δ, ρ and φ. By substituting t_z with $\tilde{t}_z(R_e, \delta, \rho, \varphi) = t_z + c(R_e, \delta, \rho, \varphi)$ and by defining

$$c(R_e, \delta, \rho, \varphi) = -\frac{1}{2\pi} \arcsin\left(\delta^{-1}\left((1-\rho)\log f - \log(R_e \|\varphi \boldsymbol{\sigma} \boldsymbol{\beta}^{(\text{eff})}\|)\right)\right), \tag{17}$$

we obtain that the new equation

$$f = R_e \exp\left\{\delta \sin\left(2\pi\left(\frac{t}{52} - \tilde{t}_z + t_s\right)\right)\right\} \|\varphi \boldsymbol{\sigma} \boldsymbol{\beta}^{(\text{eff})}\| f^{\rho} \tag{18}$$

has a solution t^* that only depends on f, t_z and t_s. Therefore by implementing the additive adjustment into the model we achieve that the beginning of the influenza season is not affected through changes in the parameters R_e, δ, ρ, and φ (and even $\boldsymbol{\sigma}$), but solely controlled by the shift parameters t_z and t_s, which considerably reduces parameter collinearity within the model.

Again note, that due to some very crude simplifications not all parameter dependencies were removed. Furthermore, we have to make some assumption on the steady state fraction f that leads to sufficient reproduction of the infection and scales the impact of ρ within the additive adjustment. While actually this fraction depends on the value of ρ itself subject to the phenomenological transmission rate, for our purposes we assumed that $f = 10^{-4}$.

For the purpose of illustration, we exemplarily investigated the impact of the seasonal shift adjustment once the model was fitted. To do so we varied the estimated population fraction φ and then simulated epidemics utilizing either the adjusted $\tilde{t}_z$ or the unadjusted t_z, respectively. see Figure S4 for the corresponding results.

From the simulated epidemics one can observe that the shift correction achieves the desired effect. For models utilizing the seasonal adjustment (left column of Figure S4) any changes in the susceptible fraction φ affect primarily the intensity of the seasonal wave, but have only negligible effects on the timing – especially the beginning – of the season. In contrast, when not employing the correction (right column) an increased φ causes an earlier epidemic in the model since the reproduction threshold inherent in the transmission rate is reached at an earlier time point within a season. However, in the real world seasonal influenza epidemics start always around the same time within a calender year regardless of intensity as can be seen in Figure S1. Thus, the correction term neutralizes the time shift effect such that the timing of the season is foremost determined through the parameter t_z and is therefore consistent over the years for each subtype.

2.3 Number of cases and I-MAARI

The transmission model given through the ODE system presented in Section 2.1 captures the hidden dynamics and the temporal development of the disease compartments. The weekly occurring number of newly infected cases and I-MAARI however are not directly given in the model. These can be derived by integrating the flow from the compartment "latently infected" ($\boldsymbol{E}$ and $\boldsymbol{E}^V$) into "infectious" ($\boldsymbol{I}$ and $\boldsymbol{I}^V$). Thus the age stratified number of cases $X_i(t)$ becoming infectious in week t is given by

$$X_i(t) = \int_t^{t+1} \gamma\left(E_i(s) + E_i^V(s)\right) ds, \tag{19}$$

for each age group $i = 1, \ldots, n_a$. Note, that this counts symptomatically as well as asymptomatically infected cases since our model captures infectiousness which includes both groups. Furthermore, not all cases $X_i(t)$

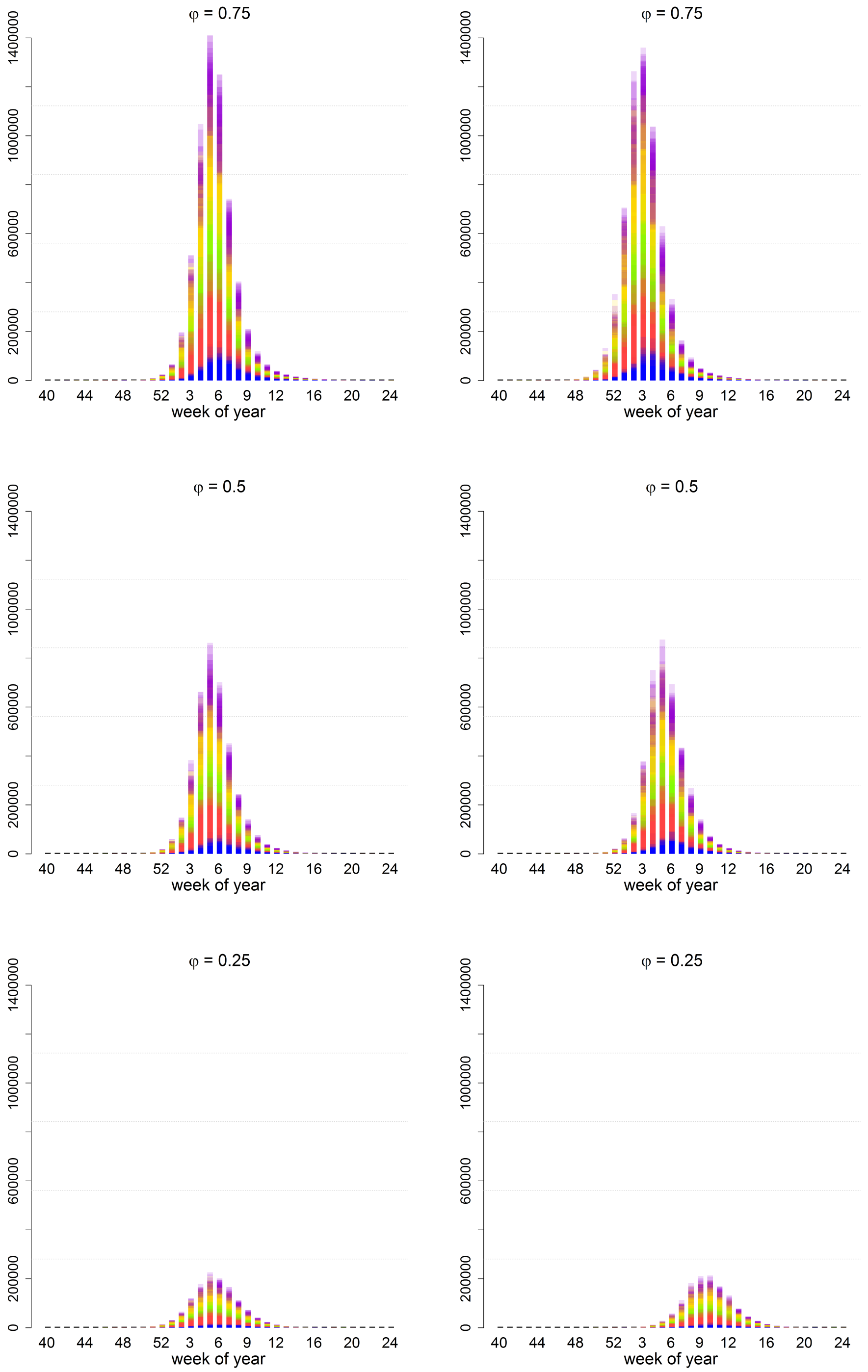

Figure S4: Comparison of simulated epidemics from transmission model using either the adjusted seasonal shift (left) or the unadjusted seasonal shift (right) for three different susceptible population fractions $\varphi \in \{0.25, 0.5, 0.75\}$.

are captured through our available data, which only consisted of counts of weekly medically attended cases attributable to influenza. Hence, in order to derive a model prediction for the weekly number of I-MAARI $Y_i(t)$ we multiply the case numbers $X_i(t)$ with a probability of developing symptoms $p^{(S)}$ and an age dependent probability $p^{(c)}(i)$ for seeking medical treatment by a general practicianer in the case of symptomatic infection, i.e.

$$Y_i(t) = p^{(S)} p^{(c)}(i) X_i(t). \tag{20}$$

The model predicted number of seasonally occurring I-MAARI will later be necessary for conducting model inference through the I-MAARI and virological data.

For the consultation probabilities $p^{(c)}(i)$ we distinguished between two age groups (<5 and 5+ years of age) which were motivated through consultation rates collected by Bayer et al. [4]. Thus, for age groups i within the age band 0-4 years we defined $p^{(c)}(i) = p_1^{(c)}$ and for all other age groups we set $p^{(c)}(i) = p_2^{(c)}$.

The probability for developing symptoms $p^{(S)}$ was fixed at 0.67 based on Carrat et al. [6]. In contrast to the consultation probabilities, the parameter $p^{(S)}$ will not be estimated within the model inference procedure. However, since the respective probabilities for developing symptoms and seeking medical treatment have the same functionality within the model, it is negligible if one of those was fixed as long as the other remained to be subject of estimation.

2.4 Seasonal stratification and model parameters

The here presented model was designed to be applied to the transmission dynamics of each single season and subtype, which was motivated by the large variability of the yearly epidemic courses with respect to magnitude of the wave and pre-dominant strain. We believe that a continuous approach in which parameters are held constant throughout the modelled time horizon – as previously implemented by many models (e.g. [29, 33, 38]) – is not capable of capturing this inter-seasonal variation and irregular pattern, since constant parameters often produce the same epidemic course for all modelled seasons.

In contrast, the completely separate treatment of each seasonal and subtype as performed by Baguelin et al. [3], Meeyai et al. [27] does not acknowledge that some aspects of influenza transmission remain constant over the years. For instance Baguelin et al. [3] estimated parameters regarding contact behaviour and ascertainment rates independently for each season and subtype, whereas these are likely consistent over the years.

For our modelling we aimed at following a hybrid approach, which allows for variability between seasons but also acknowledges the fact that the underlying pathogen, i.e. influenza virus, remains the same in each season, which implies that some transmission aspects arguably remain the same over the years.

To do so we employed an partially stratified model structure and divided all parameters considered in the transmission model into four categories: (1) parameters which are constant for each season and subtype, (2) parameters which may differ by subtype but are constant over the seasons, (3) parameters which differ by season but are equal for each subtype, and (4) parameters which may differ by both season and subtype. An alternative hybrid approach was presented by Goeyvaerts et al. [16], who employed a continuous compartment model covering several years but allowing some parameters to vary from season to season.

Table S4 gives an overview on all model parameters which were subject of estimation, whether they were stratified by season and/or subtype and their respective prior distribution within the here employed Bayesian inference framework which will be presented in the next section.

Table S4: Model parameters which are subject of estimation. Prior distributions were defined on each parameters natural scale using either logit or log transformation. Literature evidence for prior elicitation (where available) is also provided.

Parameter	Interpretation	Stratification	Prior distribution	total nr. of parameters	Source
γ	recovery rate (1/ (latently) infectious duration)	none	$\text{logit}\,\frac{\frac{1}{\gamma}-\frac{1}{7}}{\frac{1.5}{7}} \sim \mathcal{N}(0,1)$	1	[6, 27]
R_e	baseline transmissibility	none	$\text{logit}\,(R_e) \sim \mathcal{N}(0,1)$	1	(Assumption)
ρ	Spatial clustering parameter	none	$\text{logit}\,(\rho) \sim \mathcal{N}(6,0.1)$	1	[12]
λ_o	outside force of infection	none	$\log(\lambda_o) \sim \mathcal{N}(-10,0.5)$	1	(Assumption)
m	contact matrix mixing parameter	none	$\log(m-1) \sim \mathcal{N}(0,1)$	1	([37]
δ	amplitude of transmission rate seasonality	none	$\log(\delta) \sim \mathcal{N}(-10,0.5)$	1	(Assumption)
$p_i^{(c)}$	consultation rates by age	none	$\text{logit}\,(\frac{p_1^{(c)}-0.28}{0.18}) = \text{logit}\,(\frac{p_2^{(c)}-0.19}{0.19}) \sim \mathcal{N}(0,1)$	1	[4]
d	overdispersion in observations	none	$\log(d-8) \sim \mathcal{N}(0,10)$	1	(see Section 3)
σ_a	age specific susceptibility profile	by subtype	$\text{logit}\,(\sigma_a) \sim \mathcal{N}(0,1)$	10	[3]
t_z	shift in peak transmission rate	by subtype	$\text{logit}\,(t_0-0.5) \sim \mathcal{N}(0,1)$	4	(Assumption)
t_s	seasonal shift of peak transmission	by season	$\text{logit}\,(\frac{t_s}{4}+0.5) \sim \mathcal{N}(0,1)$	10	(Assumption)
φ	susceptible population fraction	by subtype and season	$\text{logit}\,(\varphi) \sim \mathcal{N}(0,1)$	40	(Assumption)

3 Bayesian inference procedure

For estimation of the overall 72 model parameters displayed in Table S4 we applied a Bayesian inference framework, such that parameters were restricted to plausible ranges if respective prior information was available or otherwise were allowed to vary more freely. For our purposes, model inference was performed for all seasons and subtypes simultaneously due to some parameter values being held constant over the seasons and/or subtypes.

Within the Bayesian setting the plausibility of a parameter combination $\boldsymbol{\theta}$ is measured through a combination of its prior probability $\pi(\boldsymbol{\theta})$ and a parameter likelihood function $f(\boldsymbol{D},\boldsymbol{P}|\boldsymbol{\theta})$ subject to the observed I-MAARI data $\boldsymbol{D}$ and subtype distribution $\boldsymbol{P}$ (see Section 1.1). This yields the eventual posterior probability $\pi(\boldsymbol{\theta}|\boldsymbol{D},\boldsymbol{P})$ of a parameter derived from the Bayesian theorem [13], i.e.

$$\pi(\boldsymbol{\theta}|\boldsymbol{D},\boldsymbol{P}) \propto f(\boldsymbol{D},\boldsymbol{P}|\boldsymbol{\theta})\pi(\boldsymbol{\theta}). \tag{21}$$

Here, we will provide some further details on prior elicitation, the likelihood function based on the influenza disease burden data, and the employed posterior sampling algorithm.

3.1 Prior elicitation

The marginal prior distribution corresponding to the single model parameters are given in Table S4. Informative prior knowledge was not available for every model parameter. For parameters that were difficult to quantify through the epidemiological literature, we assigned a vague prior securing that the parameters were restricted to a meaningful range, e.g. the susceptible population fraction each season φ was limited to the interval $[0,1]$. For parameters with available epidemiological knowledge, it was used for prior elicitation.

Regarding the recovery rate from both latent and infectious period γ we constructed a prior based on knowledge on virus shedding and other models' assumptions towards these parameters. While a study on virus shedding shows that the latent and infectious period combined might take up to 5 day [6], transmission models implemented this knowledge very differently – with infectious durations from 1.8 to 3.8 days [3, 16, 33]. Since the eventual impact of this parameter on the transmission dynamics was found to be low, in order to achieve parsimony we assumed that the latent and infectious period are of equal length $1/\gamma$ in the range 1 to 2.5 days (similar to Meeyai et al. [27]).

For the outside force of infection λ_o we chose an uninformative prior of $\log(\lambda_o) \sim \mathcal{N}(-10, 0.5)$ such that each susceptible individual is assumed to have a chance somewhere around $1/200.000$ for becoming infected from outside of the population within a given week.

Regarding the spatial clustering we assumed that the parameter ρ is near (but below) 1 such that the transmission rate within our model remains comparable to the mass-action principle employed in other influenza transmission models.

The contact mixing parameter m as applied in equation (11) was assumed to be larger than one such that the contact structure subject to sick individuals has a larger impact on the eventually employed contact matrix, since we assume that the majority of infected individuals develop symptoms [6] and therefore behave accordingly and also because symptomatically ill people likely have a higher infectiousness which additionally pronounces this contact pattern to be more relevant for transmission.

The subtype specific transmission peak shift t_z was allowed to vary within $[-0.5, 0.5]$ to allow the full range of one year.

The seasonal shifts in peak transmission t_s were allowed to only vary within $[-0.125, 0.125]$ and as it should only be able to cause minor time shifts in the seasonal occurrence of the influenza wave.

Lastly, the two consultation rate parameters $p_1^{(c)}$ and $p_2^{(c)}$ were allowed to only move within a range suggested by data from GrippeWeb [4], i.e. $p_1^{(c)} \in [0.28, 0.46]$ and $p_2^{(c)} \in [0.19, 0.38]$. The lower bounds represent the proportion of people seeking medical consultation among individuals with acute respiratory illness (ARE) whereas the upper bounds give the consultation seeking proportions among individuals with ILI symptoms according to the web survey, averaged over the seasons for both considered age groups (< 5, ≥ 5), respectively.

Finally, the joint prior density of all parameters is obtained as the product of the single priors as given in Table S4, assuming a-priori independence.

3.2 Likelihood function

The likelihood function $f(\boldsymbol{D}, \boldsymbol{P}|\boldsymbol{\theta})$ provides the probability of observing the disease burden data $(\boldsymbol{D}, \boldsymbol{P})$ subject to the model and the parameter vector $\boldsymbol{\theta}$. Here, the disease burden consists of data on the estimated numbers of I-MAARI $D_{t,a}^{(s)}$ that occurred in week t, age group a and season s, and the number of influenza-positive specimen by subtype $\boldsymbol{P}_{t,a}^{(s)} = (P_{t,a}^{(s),\text{AH1N1}}, P_{t,a}^{(s),\text{AH3N2}}, P_{t,a}^{(s),\text{B-Vic}}, P_{t,a}^{(s),\text{B-Yam}})$ also as measured in week t, age group a and season s (see Section 1.1).

Regarding the parameter likelihood $f^{(D)}(\boldsymbol{D}|\boldsymbol{\theta})$ with respect to the number of I-MAARI, we assumed that the weekly age specific number of consultation $D_{t,a}^{(s)}$ would be a random variable with expectation equal to the number of influenza attributable consultations $\bar{Y}_a^{(s)}(t)$ as predicted by the model. To obtain the prediction $\bar{Y}_a^{(s)}(t)$, we aggregated the model predictions $Y_i^{(s,z)}(t)$ over the subtype and model age group decomposition (see Section 2.3), i.e.

$$\bar{Y}_a^{(s)}(t) = \sum_{z \in \mathcal{Z}} \sum_{i \in I_a} Y_i^{(s,z)}(t). \tag{22}$$

The aggregation over the subtypes $\mathcal{Z} = \{\text{AH1N1}, \text{AH3N2}, \text{B-Vic}, \text{B-Yam}\}$ is necessary, because the subtype-stratified model yields only subtype specific I-MAARI predictions, whereas the data comprises all subtypes. Moreover, the age group decomposition in the model (indexed by i) was more refined compared to the age resolution in the data such that some age groups also had to be aggregated. Thus, I_a contains all model age group indices that are included in the data age group a. Note, that $\bar{Y}_a^{(s)}(t)$ as the final model output exclusively depends on the model parameter $\boldsymbol{\theta}$.

The number of excess consultations $D_{t,a}^{(s)}$ was then assumed to be negative binomially distributed with mean $\bar{Y}_a^{(s)}(t)$ and dispersion $d_a^{(s)}(t)$, which was defined by

$$d_a^{(s)}(t) = \begin{cases} d\left(\bar{Y}_a^{(s)}(t)\right)^{0.1} & \text{for} \quad t \in T_{\text{season}}^{(s)} \\ 1 & \text{for} \quad t \notin T_{\text{season}}^{(s)} \end{cases}, \tag{23}$$

where

$$T_{\text{season}}^{(s)} = \left\{ t \in \{-12, \ldots, 30\} \,\middle|\, \sum_{a \in \mathcal{A}} D_{t,a}^{(s)} > 0 \right\} \tag{24}$$

is the set of weeks in a given season (s) for which the total number of estimated I-MAARI is above zero. Thus, these are the weeks that belong to the epidemic season of each given year.

The reasoning for defining two different dispersion rates lies in the nature of the I-MAARI data, which was estimated as the excess MAARI incidence within the epidemic season of influenza. Thus, excess consultations are set to zero be default for each week outside of the epidemic season, although influenza cases certainly occur in the off-season as well.

To acknowledge this fact, during model fitting the model predicted I-MAARI are allowed to deviate stronger from these zero counts during the off-season, which is implemented through the dispersion parameter being $d_a^{(s)}(t) = 1$ for those weeks in which the data suggest zero I-MAARI, whereas the model predicted I-MAARI are considerably above zero.

For weeks $t \in T_{\text{season}}^{(s)}$ within the epidemic season the dispersion parameter is constructed such that the observations $D_{t,a}^{(s)}$ have a variance of

$$\mathrm{Var}(D_{t,a}^{(s)}) = \bar{Y}_a^{(s)}(t) \left(1 + d^{-1} \left(\bar{Y}_a^{(s)}(t)\right)^{0.9}\right), \tag{25}$$

with d being a model parameter to be estimated. This definition of the dispersion appears to be somewhat arbitrary but we found that by defining $d_a^{(s)}(t) = d$ (i.e. a standard negative binomial distribution) the model had problems to fit the peaks of the influenza waves, because in that case the standard deviation of the observations would scale proportionally with the predicted mean which led to excessive overestimation of the I-MAARI data through the model.

Assuming independence between age group specific and weekly observations leads to the overall likelihood $f^{(D)}$ with respect to I-MAARI data $\boldsymbol{D}$ given through

$$f^{(D)}(\boldsymbol{D}|\boldsymbol{\theta}) = \prod_{s \in \mathcal{S}} \prod_{a \in \mathcal{A}} \prod_{t=-12}^{30} p_{\text{NegBin}} \left(D_{t,a}^{(s)} \middle| \bar{Y}_a^{(s)}(t), d_a^{(s)}(t) \right), \tag{26}$$

where p_{NegBin} denotes the probability mass function of the negative binomial distribution and $\mathcal{A}$ is the set of the five age groups considered in the I-MAARI data (Section 1.1).

Additionally to the I-MAARI data, which informs the model on the magnitude of each seasonal epidemic, we fitted the model to virological data in order to also reproduce the observed seaonal subtype distribution (Section 1.1). To construct a likelihood function $f^{(P)}(\boldsymbol{P}|\boldsymbol{\theta})$ for observing the virological data $\boldsymbol{P}$ we assumed that each influenza case seeking consultation would have the same chance of leading to a influenza positive test, regardless of which subtype caused the illness. This implies that the chance for ordering laboratory case diagnostic and test sensitivity are equal for all influenza subtypes. This leads to a Dirichlet-multinomial distribution of the weekly number of influenza positive tests by subtype for each age group. Here, the total number of draws $\bar{P}_{t,a}^{(s)}$ is determined through the total number of positive tests in the data, i.e.

$$\bar{P}_{t,a}^{(s)} = \sum_{z \in \mathcal{Z}} P_{t,a}^{(s,z)}. \tag{27}$$

The model predicted probabilities $p_{t,a}^{(s,z)}$ that one of the positive tests confirms a specific subtype $z \in \mathcal{Z}$ are drawn from a Dirichlet distribution with parameter $\boldsymbol{\alpha}$ given through the predicted number of I-MAARI for

each subtype $Y_a^{(s,z)}(t)$. Thus, the likelihood of observing a certain subtype distribution $\boldsymbol{P}_{t,a}^{(s)}$ in a given week t and age group a can be written as

$$f_{s,t,a}^{(P)}\left(\boldsymbol{P}_{t,a}^{(s)} \,\middle|\, Y_a^{(s,z)}(t), z \in \mathcal{Z}\right) = \frac{\bar{P}_{t,a}^{(s)}!}{\prod_{z\in\mathcal{Z}}\left(\boldsymbol{P}_{t,a}^{(s,z)}!\right)} \frac{\Gamma(\bar{Y}_a^{(s)}(t))}{\Gamma(\bar{P}_{t,a}^{(s)} + \bar{Y}_a^{(s)}(t))} \prod_{z\in\mathcal{Z}} \frac{\Gamma(P_{t,a}^{(s,z)} + Y_a^{(s,z)}(t))}{\Gamma(Y_a^{(s,z)}(t))}, \tag{28}$$

where $\Gamma(\cdot)$ denotes the gamma function. Again, note that the quantities $Y_a^{(s,z)}(t)$ and $\bar{Y}_a^{(s)}(t)$ are those derived by the model and thus depend on the parameter vector $\boldsymbol{\theta}$. Hence, the overall likelihood subject to the virological data is given by

$$f^{(P)}(\boldsymbol{P}|\boldsymbol{\theta}) = \prod_{s\in\mathcal{S}} \prod_{a\in\mathcal{A}} \prod_{t=-12}^{30} f_{s,t,a}^{(P)}\left(\boldsymbol{P}_{t,a}^{(s)} \,\middle|\, Y_a^{(s,z)}(t), z \in \mathcal{Z}\right) \tag{29}$$

Finally, we defined the full likelihood by assuming the two data sources to be conditionally independent. Hence, we obtain

$$f(\boldsymbol{D},\boldsymbol{P}|\boldsymbol{\theta}) = f^{(D)}(\boldsymbol{D}|\boldsymbol{\theta}) f^{(P)}(\boldsymbol{P}|\boldsymbol{\theta}). \tag{30}$$

3.3 Posterior sampling

Drawing a representative sample from the posterior distribution $\pi(\boldsymbol{\theta}|\boldsymbol{D},\boldsymbol{P})$ as introduced in Section 3 was conducted by applying an adaptive Metropolis-Hastings algorithm. This type of Markov chain Monte Carlo (MCMC) algorithm was originally presented by Haario et al. [17] and has already been applied for Bayesian inference of disease transmission models by Baguelin et al. [3] and Weidemann et al. [39].

The algorithm as applied for our model is given by Algorithm 1. The idea of this MCMC procedure is to construct suitable proposal distributions on the fly, with the aim of having simultaneously high acceptance rates and large jumps while searching through the parameter space. The algorithm requires an initial parameter candidate $\boldsymbol{\theta}_0$ – suitably already within a high posterior density region – and a covariance matrix $\boldsymbol{\Sigma}_0$ for the initial Gaussian proposal distribution.

To obtain an initial parameter vector $\boldsymbol{\theta}_0$ we performed an sequential optimization procedure consisting of alternating Nelder-Mead and gradient-based optimization algorithms in order to find a high posterior parameter vector [30].

Once we obtained a parameter vector $\boldsymbol{\theta}_0$ sufficiently close to the posterior mode, we computed the initial proposal covariance matrix by computing the component wise (approximate) second derivatives of the log posterior at $\boldsymbol{\theta}_0$, i.e. we set

$$\begin{aligned}\boldsymbol{\Sigma}_0 &= n^{-1}\,\mathrm{Diag}\left(-\left(\frac{\log\pi(\boldsymbol{\theta}_0 + he_i|\boldsymbol{D},\boldsymbol{P}) - 2\log\pi(\boldsymbol{\theta}_0|\boldsymbol{D},\boldsymbol{P}) + \log\pi(\boldsymbol{\theta}_0 - he_i|\boldsymbol{D},\boldsymbol{P})}{h^2}\right)^{-1}_{i=1,\dots,n}\right)\\ &\approx n^{-1}\,\mathrm{Diag}\left(-\left(\frac{\partial^2\log\pi(\boldsymbol{\theta}_0|\boldsymbol{D},\boldsymbol{P})}{\partial\theta_i^2}\right)^{-1}_{i=1,\dots,n}\right),\end{aligned} \tag{31}$$

where e_i denotes the i-th canonical unit vector, which has a one as its i-th component and zeros otherwise, h is a sufficiently small number and $n = \dim(\boldsymbol{\theta}_0)$ denotes the number of parameters in the model. This construction yields a proposal matrix which allows large jumps in (component wise) directions yielding only minor changes of the posterior and otherwise it produces small step sizes for components which have a large impact on the

Algorithm 1: Adaptive Metropolis-Hastings

Input: $\pi(\boldsymbol{\theta}\,|\boldsymbol{D},\boldsymbol{P})$: (unnormalized) posterior density
Input: $\boldsymbol{\Sigma}_0$: initial covariance matrix of the proposal distribution
Input: s_n: scaling factor for the sample covariance matrix
Input: $\boldsymbol{\theta}^{(0)}$: initial parameter value of the chain
Input: J: length of the chain
Input: K: length of the initial period
Output: $\boldsymbol{\Theta} = (\boldsymbol{\theta}_j)_{j=1,\ldots,J}$: sample from the posterior distribution
for $j = 1$ **to** J **do**

1. Set the Gaussian proposal density q_j to be

$$q_j(\boldsymbol{\theta},\boldsymbol{\theta}^*) = \phi_{\boldsymbol{\theta},\Sigma_j}(\boldsymbol{\theta}^*)$$

where ϕ is the multivariate normal density with

$$\Sigma_j = \begin{cases} \Sigma_0 & \text{, if } j \le K \\ s_d\widehat{\text{Cov}}\left(\boldsymbol{\theta}^{(0)},\ldots,\boldsymbol{\theta}^{(j-1)}\right) & \text{, if } j > K \end{cases}$$

where $\widehat{\text{Cov}}\left(\boldsymbol{\theta}^{(0)},\ldots,\boldsymbol{\theta}^{(j-1)}\right)$ denotes to the empirical covariance and s_n is a predefined scaling factor.

2. Generate a candidate vector $\boldsymbol{\theta}^*$ using the proposal distribution $q_j(\boldsymbol{\theta}^{(j-1)},\cdot)$

3. Compute the acceptance probability $\alpha = A\left(\boldsymbol{\theta}^{(j-1)},\boldsymbol{\theta}^*\right)$ with

$$A\left(\boldsymbol{\theta}^{(j-1)},\boldsymbol{\theta}^*\right) = \min\left\{1, \frac{\pi(\boldsymbol{\theta}^*\,|\boldsymbol{D},\boldsymbol{P})}{\pi\left(\boldsymbol{\theta}^{(j-1)}\,|\boldsymbol{D},\boldsymbol{P}\right)}\right\}$$

4. Set $\boldsymbol{\theta}^{(j)} = \boldsymbol{\theta}^*$ with probability α and $\boldsymbol{\theta}^{(j)} = \boldsymbol{\theta}^{(j-1)}$ otherwise

end

log posterior. This induces an overall suitable initial moving through the parameter space until the adaptive phase sets in. Note, that computation of the full inverse Hessian at $\boldsymbol{\theta}_0$ which would be an optimal candidate as proposal covariance is not feasible in this high-dimensional (n=72) setting.

As a remark, all parameters in $\boldsymbol{\theta}$ were transformed onto the whole real axis using appropriate log or logit transformations. For the exact choice of transformation of each parameter, please refer to the transformation used for constructing the respective prior densities as given in Table S4.

For the scaling coefficient we used $s_n = 2.4^2/n$ as recommended by [14] in order to achieve a suitable acceptance rate for the MCMC algorithm.

3.4 Inference results

Posterior means (together with 95%-prediction intervals) of each estimated model parameter are given in Table 1 of the main article, a graphical overview based on kernel density estimates is provided by Figure S5. The posterior distributions of the susceptible population fractions depending on age group, subtype, and year are displayed by Figure 2 within the main article.

4 Sensitivity analyses

In order to investigate the robustness of the vaccination impact results, we examined the impact of selected alternative model features. These include the assumption of mass-action transmission, the suppression of indirect effects, not accounting for an adjusted contact matrix due to symptomatic illness, and the assumption of a B-lineage vaccine cross protection of 0%. Within this section we present the construction of each alternative model version and its associated impact prediction regarding a childhood vaccination program as provided in Table 3 of the main article.

Each presented model version was fitted to the same data on I-MAARI and subtype distribution using the same inference procedures given in Section 3. The validity of each model was measured through its associated marginal loglikelihood of the data given in Table 3 of the main article [19]. Marginal loglikelihood differences larger than 5 indicate a very strong evidence for the model yielding the higher likelihood. Nevertheless, the marginal likelihood should be interpreted with caution, since it only assesses the likelihood of observing the data subject to a specific model but it does account for the plausibility of the underlying model.

4.1 Model version: mass-action-transmission

Within the first alternative model we omitted the power parameter ρ within the phenomenological transmission rate of the original model. In other words, we set $\rho = 1$ and the transmission rate becomes

$$\lambda_i(t, \boldsymbol{I}, \boldsymbol{I}^V) = R_e \gamma \exp\left\{\delta \sin\left(2\pi\left(\frac{t}{52} - t_z + t_s\right)\right)\right\} \sum_{j=1}^{n_a} \beta_{ji}^{(\mathrm{eff})} \frac{I_j + I_j^V}{N_j} + \lambda_o, \tag{32}$$

which corresponds to the classical mass-action-principle as it was applied in all other influenza transmission models [3, 16, 27, 29, 33, 38]. The fitting and the corresponding vaccination impact results subject to this mass-action-transmission model are given in Figures S6 and S7, respectively.

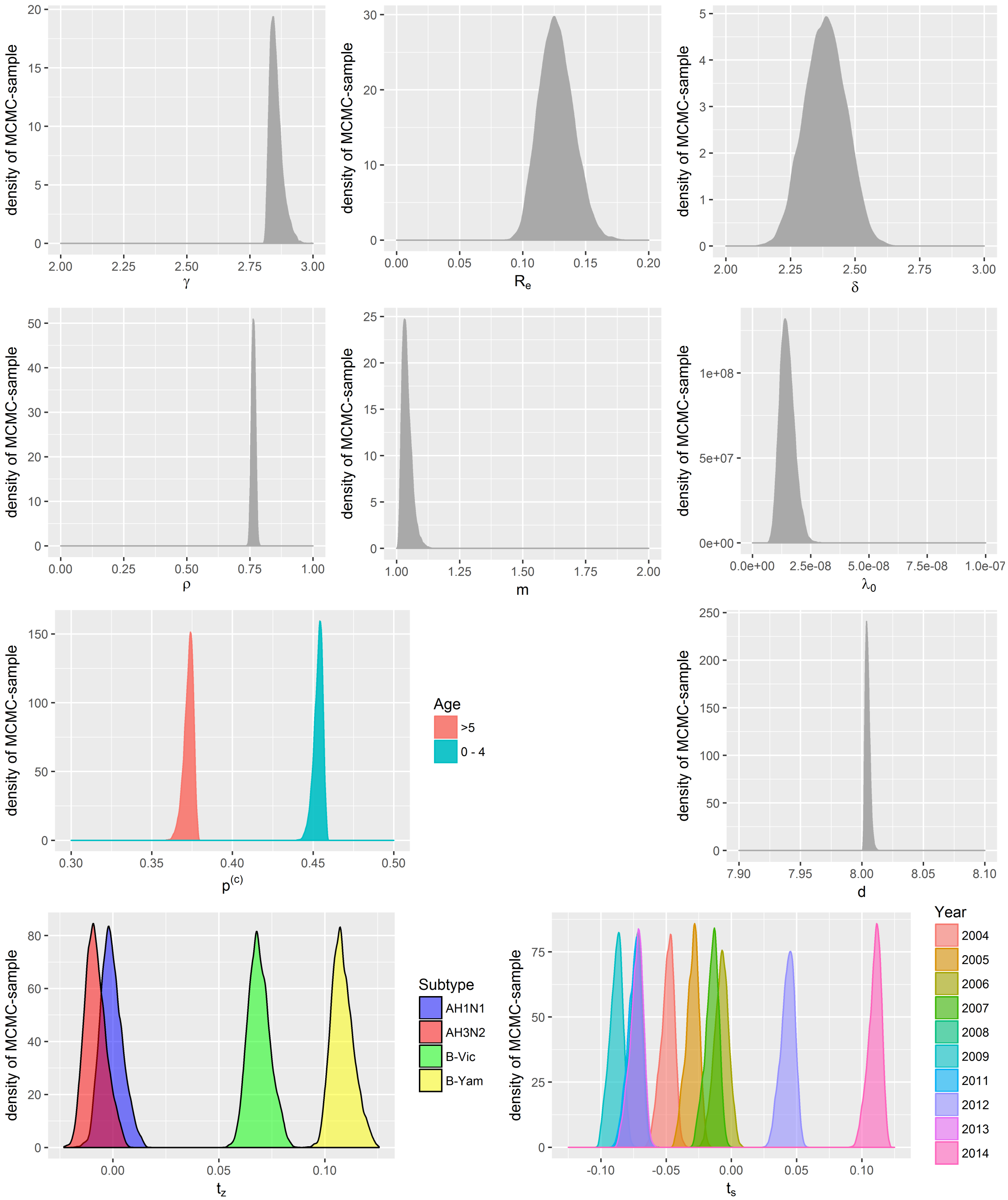

Figure S5: Kernel density estimate of each model parameters posterior distribution subject to the computed posterior sample using MCMC. Please refer to Table 1 of the main article or Table S4 in the supplementary document for the interpretation of each displayed parameter.

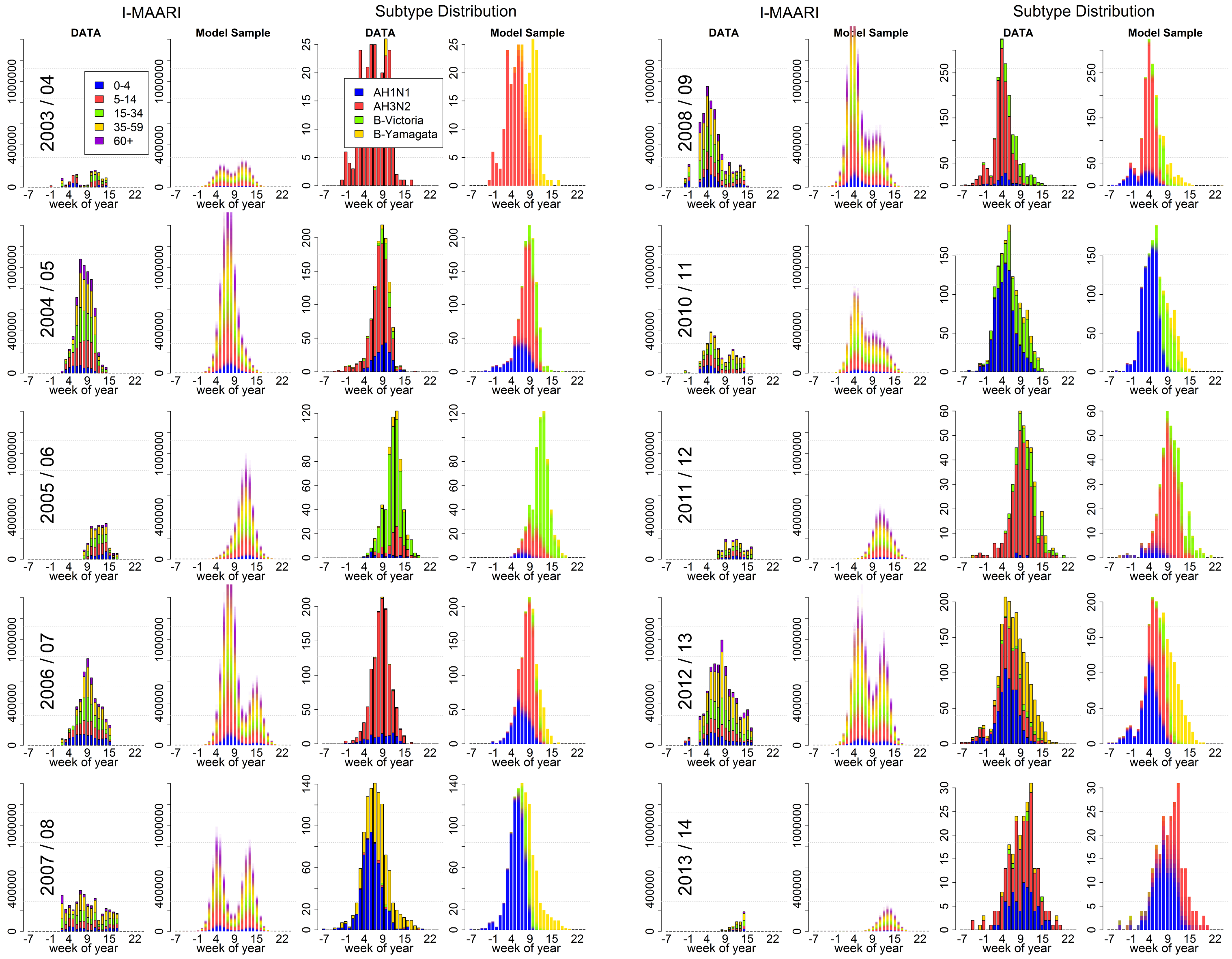

Figure S6: Influenza waves according to data and the fitted mass-action-transmission model for the seasons 2003/04 till 2013/14 (excluding the pandemic season 2009/10). The first and second column show the I-MAARI data and corresponding model-predicted counts, respectively. The third and fourth column provide virological data and corresponding model-predicted subtype distribution, respectively. Model predictions are displayed based on overlaid output subject to 200 parameter vectors drawn from the posterior distribution.

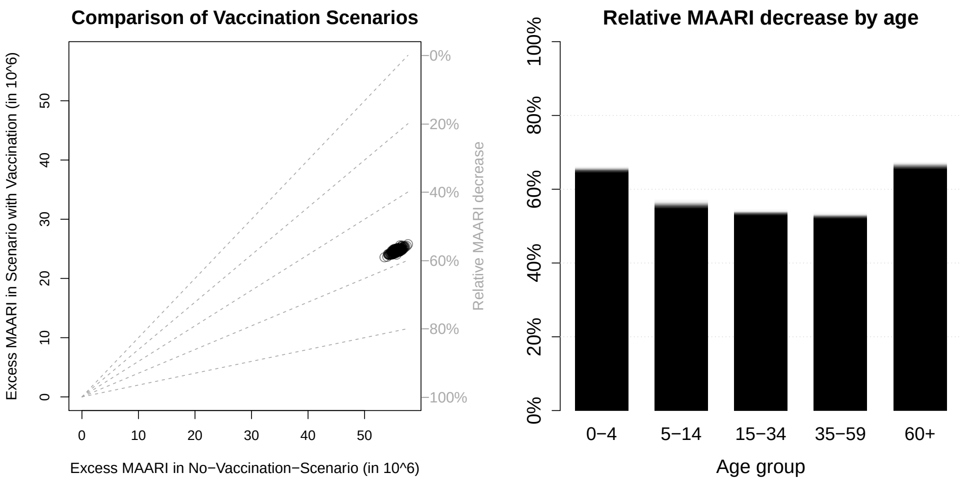

Figure S7: Relative impact of childhood vaccination (40% coverage among 2-10 year old children) based on a model scenario assuming mass-action-transmission.

4.2 Model version: disabled indirect effects

In this model scenario vaccination does not protect against infection but against illness instead. Thus, this model is not capable of generating any indirect vaccination effects, since vaccinated and unvaccinated people are equally likely to obtain infection and transmit the virus. This is implemented by adjusting the initial conditions of the ODE system (Section 2.1.1), i.e. in this model version we set

$$\begin{aligned}
S_i(t_0) &= \varphi^{(s,z)}\sigma_{a_i}^{(s,z)}(1-\mathrm{VC}_i^{(s)})N_i & S_i^V(t_0) &= \varphi^{(s,z)}\sigma_{a_i}^{(s,z)}\mathrm{VC}_i^{(s)}N_i \\
E_i(t_0) &= 0 & E_i^V(t_0) &= 0 \\
I_i(t_0) &= 0 & I_i^V(t_0) &= 0 \\
R_i(t_0) &= (1-\varphi^{(s,z)}\sigma_{a_i}^{(s,z)})(1-\mathrm{VC}_i^{(s)})N_i & R_i^V(t_0) &= \left(1-\varphi^{(s,z)}\sigma_{a_i}^{(s,z)}\right)\mathrm{VC}_i^{(s)}N_i. \qquad (33)
\end{aligned}$$

Additionally, for this model scenario the proportion of cases leading to consultation are modified in order to distinguish between vaccinated cases

$$X_i^V(t) = \int_t^{t+1} \gamma E_i^V(s)\,ds, \qquad (34)$$

and unvaccinated cases

$$X_i(t) = \int_t^{t+1} \gamma E_i(s)\,ds, \qquad (35)$$

for each age group i. The modified number of resulting consultations per season and subtype then accounted for the vaccine protection, i.e.

$$Y_i(t) = p^{(S)}p^{(c)}(i)\left(X_i(t) + (1-\mathrm{VE}_i)X_i^V(t)\right). \qquad (36)$$

The predicted childhood vaccination impact subject to this model is given in Figure S8.

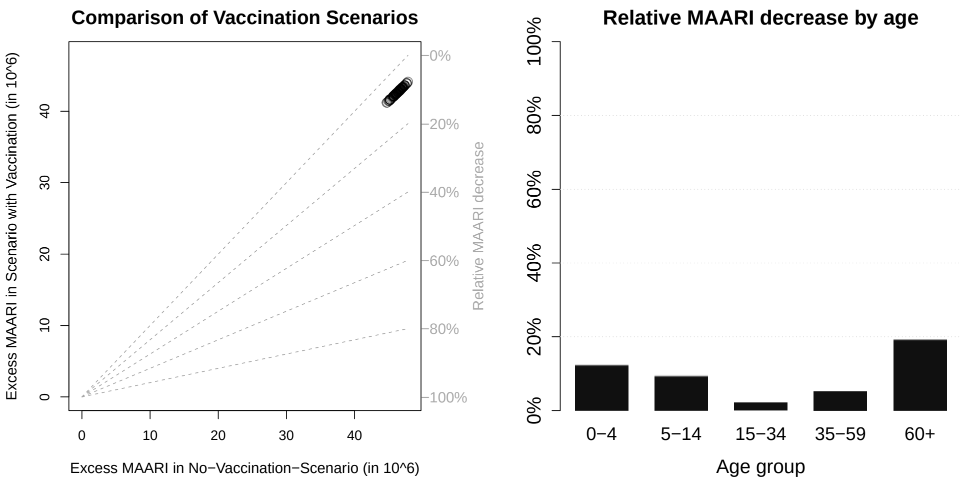

Figure S8: Relative impact of childhood vaccination (40% coverage among 2-10 year old children) based on a model scenario disabling indirect vaccination effects.

4.3 Model version: POLYMOD contact matrix

For the third model scenario we dropped the assumption of a different contact pattern subject to symptomatic illness as proposed by Van Kerckhove et al. [37] and used solely the POLYMOD matrix subject to healthy individuals (Section 1.5). Thus, the model contact matrix is given by

$$\beta^{(\text{eff})} = \nu^{-1}\beta^{(\text{healthy})}, \tag{37}$$

where ν refers to the maximum eigenvalue of $\beta^{(\text{healthy})}$. Hence, the mixing parameter m is omitted in this model scenario. Vaccination impact subject to this model are given in Figure S9.

4.4 Model version: no B-lineage cross protection

This model scenario utilized a different VE matrix as compared to the VEs given in Table S3. Differences occur by assuming 0% vaccine effectiveness against the B-lineage that is not included in the vaccine according to WHO recommendation. This affects the VE estimates that are coloured red in Table S3. The corresponding vaccination impact results are given in Figure S10.

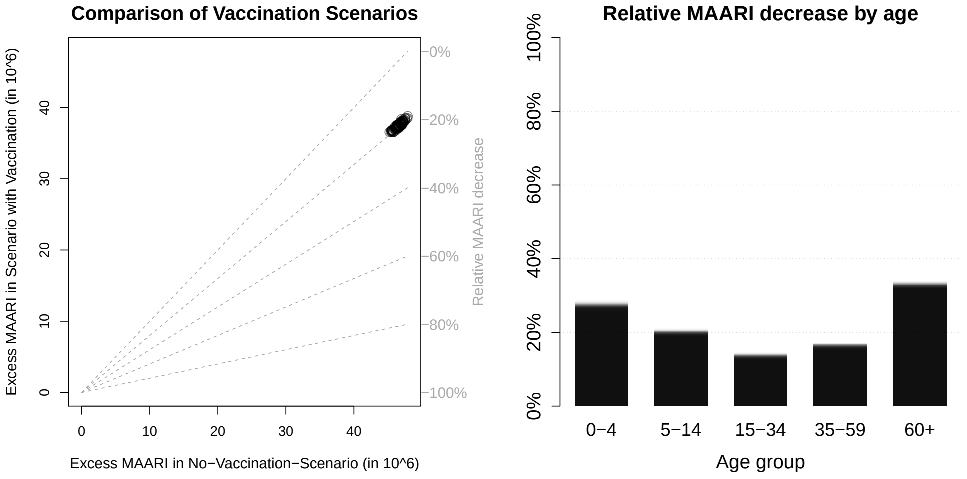

Figure S9: Relative impact of childhood vaccination (40% coverage among 2-10 year old children) based on a model scenario utilizing only the POLYMOD contact matrix.

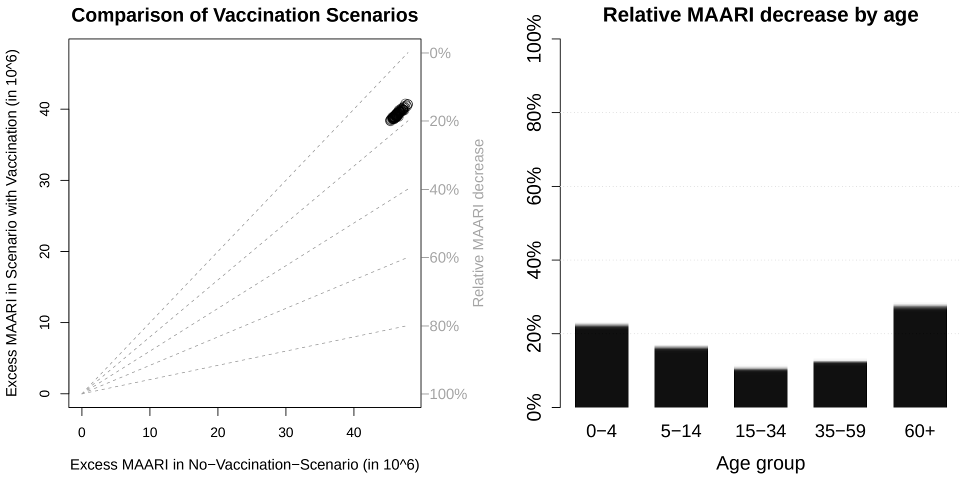

Figure S10: Relative impact of childhood vaccination (40% coverage among 2-10 year old children) based on a model scenario assuming no B-lineage crossprotection.

References

[1] an der Heiden, M., Köpke, K., Buda, S., Buchholz, U., and Haas, W. (2013). Estimates of Excess Medically Attended Acute Respiratory Infections in Periods of Seasonal and Pandemic Influenza in Germany from 2001/02 to 2010/11. *PLoS ONE*, 8(7):e64593.

[2] Anderson, R. M. and May, R. M. (1991). *Infectious Diseases of Humans: Dynamics and Control*. Oxford University Press.

[3] Baguelin, M., Flasche, S., Camacho, A., Demiris, N., Miller, E., and Edmunds, W. J. (2013). Assessing optimal target populations for influenza vaccination programmes: An evidence synthesis and modelling study. *PLoS Med*, 10(10):e1001527.

[4] Bayer, C., Remschmidt, C., an der Heiden, M., Tolksdorf, K., Herzhoff, M., Kaersten, S., Buda, S., Haas, W., and Buchholz, U. (2014). Internet-based syndromic monitoring of acute respiratory illness in the general population of Germany, weeks 35/2011 to 34/2012. *Euro Surveill.*, 19(4):pii=20684.

[5] Birrell, P. J., Ketsetzis, G., Gay, N. J., Cooper, B. S., Presanis, A. M., Harris, R. J., Charlett, A., Zhang, X.-S., White, P. J., Pebody, R. G., and De Angelis, D. (2011). Bayesian modeling to unmask and predict influenza A/H1N1pdm dynamics in London. *Proceedings of the National Academy of Sciences*, 108(45):18238–18243.

[6] Carrat, F., Vergu, E., Ferguson, N. M., Lemaitre, M., Cauchemez, S., Leach, S., and Valleron, A.-J. (2008). Time lines of infection and disease in human influenza: A review of volunteer challenge studies. *American Journal of Epidemiology*, 167(7):775–785.

[7] Chowell, G., Viboud, C., Simonsen, L., and Moghadas, S. M. (2016). Characterizing the reproduction number of epidemics with early subexponential growth dynamics. *Journal of The Royal Society Interface*, 13(123).

[8] Demicheli, V., Jefferson, T., Al-Ansary, L. A., Ferroni, E., Rivetti, A., and Di Pietrantonj, C. (2014). Vaccines for preventing influenza in healthy adults. *Cochrane Database of Systematic Reviews*, (3).

[9] Eames, K., Tilston, N., White, P., Adams, E., and Edmunds, J. (2010). The impact of illness and the impact of school closure on social contact patterns. *Health Technology Assessment*, 14.

[10] Eichner, M., Schwehm, M., Hain, J., Uphoff, H., Salzberger, B., Knuf, M., and Schmidt-Ott, R. (2014). 4flu - an individual based simulation tool to study the effects of quadrivalent vaccination on seasonal influenza in germany. *BMC Infectious Diseases*, 14(1):1–21.

[11] Federal Bureau of Statistics (2015). GENESIS Online Database.

[12] Finkenstdt, B. F. and Grenfell, B. T. (2000). Time series modelling of childhood diseases: a dynamical systems approach. *Journal of the Royal Statistical Society: Series C (Applied Statistics)*, 49(2):187–205.

[13] Gelman, A., Carlin, J., Stern, H., Dunson, D., Vehtari, A., and D., R. (2013). *Bayesian Data Analysis*. CRC Press, New Jersey.

[14] Gelman, A., Roberts, G. O., and Gilks, W. R. (1996). Efficient Metropolis jumping rules. *Bayesian statistics*, 5:599–607.

[15] Goeyvaerts, N., Hens, N., Ogunjimi, B., Aerts, M., Shkedy, Z., Van Damme, P., and Beutels, P. (2010). Estimating infectious disease parameters from data on social contacts and serological status. *Journal of the Royal Statistical Society. Series C (Applied Statistics)*, 59(2):255–277.

[16] Goeyvaerts, N., Willem, L., Kerckhove, K. V., Vandendijck, Y., Hanquet, G., Beutels, P., and Hens, N. (2015). Estimating dynamic transmission model parameters for seasonal influenza by fitting to age and season-specific influenza-like illness incidence. *Epidemics*, 13:1 – 9.

[17] Haario, H., Saksman, E., and Tamminen, J. (2001). An Adaptive Metropolis algorithm. *Bernoulli*, 7:223–242.

[18] Jefferson, T., Rivetti, A., Di Pietrantonj, C., Demicheli, V., and Ferroni, E. (2012). Vaccines for preventing influenza in healthy children. *Cochrane Database of Systematic Reviews*, (8).

[19] Kass, R. E. and Raftery, A. E. (1995). Bayes factors. *Journal of the American Statistical Association*, 90(430):773–795.

[20] Kissling, E., Valenciano, M., Buchholz, U., Larrauri, A., Cohen, J. M., Nunes, B., Rogalska, J., Pitigoi, D., Paradowska-Stankiewicz, I., Reuss, A., Jimnez-Jorge, S., Daviaud, I., Guiomar, R., ODonnell, J., Necula, G., Guchowska, M., and Moren, A. Influenza vaccine effectiveness estimates in Europe in a season with three influenza type/subtypes circulating: the I-MOVE multicentre casecontrol study, influenza season 2012/13. Euro Surveill. 2014;19(6):pii=20701. Article DOI: http://dx.doi.org/10.2807/1560-7917.ES2014.19.6.20701.

[21] Kissling, E., Valenciano, M., Cohen, J. M., Oroszi, B., Barret, A.-S., Rizzo, C., Stefanoff, P., Nunes, B., Pitigoi, D., Larrauri, A., Daviaud, I., Horvath, J. K., O'Donnell, J., Seyler, T., Paradowska-Stankiewicz, I. A., Pechirra, P., Ivanciuc, A. E., Jimnez-Jorge, S., Savulescu, C., Ciancio, B. C., and Moren, A. (2011). I-move multi-centre case control study 2010-11: Overall and stratified estimates of influenza vaccine effectiveness in europe. *PLoS ONE*, 6(11):e27622.

[22] Kissling, E., Valenciano, M., Falco, J. M., Larrauri, A., Widgren, K., Pitigoi, D., Oroszi, B., Nunes, B., Savulescu, C., Mazick, A., Lupulescu, E., Ciancio, B., and Moren, A. I-MOVE towards monitoring seasonal and pandemic influenza vaccine effectiveness: lessons learnt from a pilot multi-centric case-control study in Europe, 2008-9. Euro Surveill. 2009;14(44):pii=19388. Available online: http://www.eurosurveillance.org/ViewArticle.aspx?ArticleId=19388.

[23] Kissling, E., Valenciano, M., Larrauri, A., Oroszi, B., Cohen, J. M., Nunes, B., Pitigoi, D., Rizzo, C., Rebolledo, J., Paradowska-Stankiewicz, I., Jimnez-Jorge, S., Horvth, J. K., Daviaud, I., Guiomar, R., Necula, G., Bella, A., ODonnell, J., Guchowska, M., Ciancio, B. C., Nicoll, A., and Moren, A. Low and decreasing vaccine effectiveness against influenza A(H3) in 2011/12 among vaccination target groups in Europe: results from the I-MOVE multicentre casecontrol study. Euro Surveill. 2013;18(5):pii=20390. Available online: http://www.eurosurveillance.org/ViewArticle.aspx?ArticleId=20390.

[24] Kong, L., Wang, J., Han, W., and Cao, Z. (2016). Modeling heterogeneity in direct infectious disease transmission in a compartmental model. *International Journal of Environmental Research and Public Health*, 13(3):253.

[25] Langley, J. M., Carmona Martinez, A., Chatterjee, A., Halperin, S. A., McNeil, S., Reisinger, K. S., Aggarwal, N., Huang, L.-M., Peng, C.-T., Garcia-Sicilia, J., Salamanca de la Cueva, I., Cabaas, F., Trevio-Garza, C., Rodrguez-Weber, M. A., de la O, M., Chandrasekaran, V., Dew, W., Liu, A., Innis, B. L., and

Jain, V. K. (2013). Immunogenicity and safety of an inactivated quadrivalent influenza vaccine candidate: A phase iii randomized controlled trial in children. *Journal of Infectious Diseases*, 208(4):544–553.

[26] McCallum, H., Barlow, N., and Hone, J. (2001). How should pathogen transmission be modelled? *Trends in Ecology & Evolution*, 16(6):295 – 300.

[27] Meeyai, A., Praditsitthikorn, N., Kotirum, S., Kulpeng, W., Putthasri, W., Cooper, B. S., and Teerawattananon, Y. (2015). Seasonal influenza vaccination for children in thailand: A cost-effectiveness analysis. *PLoS Med*, 12(5):e1001829.

[28] Mossong, J., Hens, N., Jit, M., Beutels, P., Auranen, K., Mikolajczyk, R., Massari, M., Salmaso, S., Tomba, G. S., Wallinga, J., Heijne, J., Sadkowska-Todys, M., Rosinska, M., and Edmunds, W. J. (2008). Social Contacts and Mixing Patterns Relevant to the Spread of Infectious Diseases. *PLoS Med*, 5(3):e74.

[29] Pitman, R., White, L., and Sculpher, M. (2012). Estimating the clinical impact of introducing paediatric influenza vaccination in england and wales. *Vaccine*, 30(6):1208 – 1224.

[30] Press, W. H., Teukolsky, S. A., Vetterling, W. T., and Flannery, B. P. (2007). *Numerical Recipes 3rd Edition*. Cambridge University Press.

[31] Rieck, T., Feig, M., Eckmanns, T., Benzler, J., Siedler, A., and Wichmann, O. (2014). Vaccination coverage among children in germany estimated by analysis of health insurance claims data. *Human Vaccines & Immunotherapeutics*, 10(2):476–484. PMID: 24192604.

[32] Robert Koch-Institut (2014). Bericht zur Epidemiologie der Influenza in Deutschland. Saison 2013/14. https://influenza.rki.de/Saisonbericht.aspx.

[33] Rose, M. A., Damm, O., Greiner, W., Knuf, M., Wutzler, P., Liese, J. G., Krüger, H., Wahn, U., Schaberg, T., Schwehm, M., Kochmann, T. F., and Eichner, M. (2014). The epidemiological impact of childhood influenza vaccination using live-attenuated influenza vaccine (laiv) in germany: predictions of a simulation study. *BMC Infectious Diseases*, 14(1).

[34] Tricco, A. C., Chit, A., Soobiah, C., Hallett, D., Meier, G., Chen, M. H., Tashkandi, M., Bauch, C. T., and Loeb, M. (2013). Comparing influenza vaccine efficacy against mismatched and matched strains: a systematic review and meta-analysis. *BMC Medicine*, 11(1):1–19.

[35] Valenciano, M., Kissling, E., Cohen, J.-M., Oroszi, B., Barret, A.-S., Rizzo, C., Nunes, B., Pitigoi, D., Larrauri Cmara, A., Mosnier, A., Horvath, J. K., O'Donnell, J., Bella, A., Guiomar, R., Lupulescu, E., Savulescu, C., Ciancio, B. C., Kramarz, P., and Moren, A. (2011). Estimates of pandemic influenza vaccine effectiveness in europe, 20092010: Results of influenza monitoring vaccine effectiveness in europe (i-move) multicentre case-control study. *PLoS Med*, 8(1):e1000388.

[36] Valenciano, M., Kissling, E., Reuss, A., Jimnez-Jorge, S., Horvth, J. K., Donnell, J. M., Pitigoi, D., Machado, A., and Pozo, F. (2015). The european i-move multicentre 20132014 case-control study. homogeneous moderate influenza vaccine effectiveness against a(h1n1)pdm09 and heterogenous results by country against a(h3n2). *Vaccine*, 33(24):2813 – 2822.

[37] Van Kerckhove, K., Hens, N., Edmunds, W. J., and Eames, K. T. D. (2013). The impact of illness on social networks: Implications for transmission and control of influenza. *American Journal of Epidemiology*, 178(11):1655–1662.

[38] Vynnycky, E., Pitman, R., Siddiqui, R., Gay, N., and Edmunds, W. J. (2008). Estimating the impact of childhood influenza vaccination programmes in england and wales. *Vaccine*, 26(41):5321 – 5330.

[39] Weidemann, F., Dehnert, M., Koch, J., Wichmann, O., and Hhle, M. (2014). Modelling the epidemiological impact of rotavirus vaccination in Germany A Bayesian approach. *Vaccine*, 32(40):5250 – 5257.
